# *Cordyceps militaris* extract induces apoptosis and pyroptosis via caspase‐3/PARP/GSDME pathways in A549 cell line

**DOI:** 10.1002/fsn3.2636

**Published:** 2021-10-30

**Authors:** Zixuan Hu, Yijing Lai, Chaoya Ma, Lina Zuo, Guanlin Xiao, Haili Gao, Biyuan Xie, Xuejun Huang, Haining Gan, Dane Huang, Nan Yao, Baoguo Feng, JieXia Ru, Yuxing Chen, Dake Cai

**Affiliations:** ^1^ The Fifth Clinical Medical College Guangzhou University of Chinese Medicine Guangzhou China; ^2^ Guangdong Provincial Key Laboratory of Research and Development in Traditional Chinese Medicine Guangzhou China; ^3^ Guangdong Provincial Key Laboratory of Occupational Disease Prevention and Treatment Department of Science and Education Guangdong Province Hospital for Occupational Disease Prevention and Treatment Guangzhou China; ^4^ Health examination center Sun Yat‐sen Memorial Hospital, Sun Yat‐Sen University Guangzhou 510120 China; ^5^ GENETERRA (Chinese) Research Center Guangzhou China; ^6^ College of Materials and Energy South China Agricultural University Guangzhou China

**Keywords:** A549, anticancer, apoptosis, *Cordyceps militaris*, pyroptosis

## Abstract

*Cordyceps militaris* (CM) is traditionally used as dietary therapy for lung cancer patients in China. CM extract (CME) is hydrosoluble fraction of CM and extensively investigated. Caspase‐3‐involved cell death is considered as its major anticancer mechanism but inconclusive. Therefore, we explore its caspase‐3‐dependent programmed cell death nature (apoptosis and pyroptosis) and validate its caspase‐3‐dependent property in loss‐of‐function experiment. Component profile of CME is detected by High Performance Liquid Chromatography‐ quadrupole time‐of‐flight mass spectrometry (HPLC‐qTOF). Results show that CME causes pyroptosis‐featured cell bubbling and cell lysis and inhibits cell proliferation in A549 cell. CME induces chromatin condensing and makes PI+/annexin V+ staining in bubbling cells, indicating genotoxicity, apoptosis, and pyroptosis cell death are caused by CME. High concentration of CME (200 μg/ml) exerts G2/M and G0 cell cycles arresting and suppresses P53‐downstream proliferative proteins, including P53, P21, CDC25B, CyclinB1, Bcl‐2, and BCL2 associated agonist of cell death (BAD), but 1–100 μg/ml of CME show less effect on proteins above. Correspondingly, caspase‐3 activity and caspase‐3 downstream proteins including pyroptotic effector gasdermin‐E (GSDME) and apoptotic marker cleaved‐poly‐ADP‐ribose polymerase (PARP) are significantly promoted by CME. Moreover, regarding membrane pore formation in pyroptotic cell, expression of membrane GSDME (GSDME antibody conjugated with PE‐Cy7 for detection in flow cytometry) is remarkably increased by CME treatment. By contrast, other pyroptosis‐related proteins such as P2X7, NLRP3, GSDMD, and Caspase‐1 are not affected after CME treatment. Additionally, TET2 is unexpectedly raised by CME. In present of caspase‐3 inhibitor Ac‐DEVD‐CHO (Ac‐DC), CME‐induced cytotoxicity, cell bubbling, and genotoxicity are reduced, and CME‐induced upregulation of apoptosis (cleaved‐PARP‐1) and pyroptosis (GSDME‐NT) proteins are reversed. Lastly, 22 components are identified in HPLC‐qTOF experiment, and they are classified into trophism, neoadjuvant component, cytotoxic component, and cancer deterioration promoter according to previous references. Conclusively, CME causes caspase‐3‐dependent apoptosis and pyroptosis in A549 through caspase‐3/PARP and caspase‐3/GSDME pathways, and it provides basic insight into clinic application of CME for cancer patients.

## Graphical abstract

The present study has shown that the *Cordyceps militaris* extract CME exerts both apoptosis and pyroptosis toward A549 cell line through inducing caspase‐3‐depended pyroptosis and apoptosis.

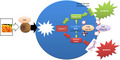

## 1 INTRODUCTION

Lung cancer is the most ubiquitous malignant with highest incidence of cancer‐related death worldwide (Hirsch et al., 2017). Nonsmall cell lung cancer (NSCLC) is one of major subtypes of lung cancer, and it accounts for 80% of lung cancers and has a low 5‐year survival rate (Huang, Li, et al., 2018). Despite that advance is made in anticancer medicine for NSCLC, side effect of first‐line chemotherapy still remains one of the barriers for sustainable therapy (Merk et al., 2011). Therefore, adjuvant therapy is suggested for maintaining efficacy and alleviating side effect in chemotherapy interval. Herbal and dietary therapies are considered as alternative option for first‐line cancer treatment due to their anticancer properties and tonic effect. *Cordyceps militaris*, a dietary food in South China, is traditionally used as adjuvant treatment for cancer patient as decoction (Cai et al., 2018). Its adjuvant usage may partially stem from its cytotoxicity toward cancer cell as emerging evidence revealed (Bai & Sheu, 2018; Chou et al., 2014; Kim et al., 2017; Lee et al., 2015). Meanwhile, adenosine, cordycepin, and cordycepic acid, which are bioactive components in *C. militaris* extract (CME), have been identified as proapoptotic constituents. However, its clinic application is still controversial due to the concern that cytotoxic effect and targets of CME are not validated. Therefore, loss‐of‐function assay is applied to validate its cytotoxicity and target gene of CME in our research, and this validation will improve our understanding on anticancer property of CME.

Cytotoxic effect and programmed cell death (PCD) are some properties of adjuvant medicine, and they are taken into consideration in their clinic applications. These properties are still elusive in herbal medicine; therefore, more precise and validated PCD mechanism is needed for convincing clinic practicians. In term of PCD regulation, caspase‐3 is a critical enzyme that integrated upstream signaling and consequently regulated cell death including apoptosis and pyroptosis. Caspase‐3‐dependent pyroptosis, which is inflammation‐involved PCD and one of the downstream pathways of reactive oxygen species (ROS), is increasingly recognized as critical pathways for PCD beyond apoptosis (Wang et al., 2017). CME is reported to induce apoptosis through affecting multiple targets (Bub et al., 2019). Noticeably, intracellular ROS production is frequently reported to be responsive molecule for proapoptotic effect of CME (Nasser et al., 2017). ROS triggers mitochondria‐ (Lee & Hong, 2011) and caspase‐3‐involved apoptosis and mammalian target of rapamycin‐involved autophagic cell death in A549 cell line. However, CME‐induced pyroptosis is not reported even though it may play a critical role in adjuvant treatment. CME‐induced pyroptosis is hypothesized in our research because that CME is also found to exert immunogenic cell death, and this kind of cell death is indicative of pyroptosis‐related effect (Han et al., 2010; Quan et al., 2020). Therefore, pyroptosis and capsase‐3‐mediating effect are chosen for validating anticancer mechanism of CME in our research. Caspase‐3 inhibitor Ac‐DEVD‐CHO (Ac‐DC) is employed to validate caspase‐3‐dependent effect and mechanism. Specifically, Ac‐DC is used to get a loss of function of caspase‐3, and regulative effect of CME is comparatively validated in A549 with inactive caspase‐3 or normal caspase‐3. In our research, CME is found to cause caspase‐3‐dependent pyroptosis and apoptosis toward NSCLC A549 cell line, and it is firstly confirmed that caspase‐3‐depended pyroptosis can be induced by CME.

Feature of pyroptosis is defined in cell morphology, including cell lysis, cell bubbling, and inflammation. Moreover, a set of proteins called gasdermin (GSDM) family have been characterized as biomarker of pyroptosis (Shi et al., 2017; Zhu et al., 2018), and these proteins could be activated by ROS/inflammasome through caspase‐depended pathway (Zhang et al., 2019) in lung cancer. In term of inflammation in pyroptosis, interleukin‐1 beta (IL‐1β) is also stimulated and excreted through GSDM‐formed membrane pore, and it is regulated by P2X purinoceptor 7 (P2X7R)/tet methylcytosine dioxygenase 2 (TET2)/NLR family pyrin domain containing 3 (NLRP3), which is another downstream pathway of ROS. Proinflammatory nature, cell lysis, and marker proteins GSDM not only cause distinguished cell death but also sensitize cancer cells to immunity system (Jorgensen et al., 2016; Kovacs & Miao, 2017), and it may provide new insight into clinic application of CME as an adjuvant treatment. In sum, feature of CME‐induced pyroptosis is explored and validated in phenotype and genotype as described above. Fortunately, slight CME‐induced pyroptosis is validated in loss of function of caspase‐3 even though its clinic application needs further refinement of CME which can achieve greater efficacy, and our finding highlights rationale of clinic application of CME as a dietary therapy.

## 2 METHOD

### 2.1 Material

*Cordyceps militaris* extract (CME) is purchased from GENETERRA (CHINESE) CO. LTD. Contents of cordycepin, adenosine, and crude polysaccharideare are 3749.29 ± 36.90 mg/kg, 4022.26 ± 13.58 mg/kg, and 27.4 ± 2.83 g/kg, respectively, in CME by high‐performance liquid chromatography (HPLC; Appendix S1). Crude polysaccharideare was detected according to previous method (Qiao et al., 2019).

### 2.2 Cell culture and treatment

A549 was purchased from the Shanghai Institute of Cell Biology and was cultured using Dulbecco's Modified Eagle's medium (DMEM, Gibco) supplemented with 10% fetal bovine serum (FBS) (Bovogen), 100 U/ml penicillin, and 100 μg/ml streptomycin (Gibco) in 37℃ in incubator with 5% CO_2_ saturation.

### 2.3 Cell cytotoxicity

Cytotoxicity of CME on A549 cells was measured by using the Cell Counting Kit‐8 (CCK‐8, Dojindo) as previously described (Zhang et al., 2016) with modification. Briefly, cells were seeded in 96‐well plates at a density of 1 × 10^4^ cells/well and treated with different concentrations of CME (0, 1, 10, 100, 200, 400, 600, 800, and 1000 μg/ml) for 24, 48, or 72 h, and cells were observed by using microscope before addition of CCK‐8. CCK‐8 solution was added to each well and incubated for another 1 h. The absorbance was measured using a microplate reader (Varioskan Flash, Thermo) at 450 nm.

### 2.4 Annexin V‐FITC and propidium iodide assay

Cell apoptosis was analyzed using the Annexin V‐FITC Apoptosis Detection Kit (Beyotime) according to the manufacturer's instructions. Briefly, A549 cells were seeded in 6‐well plates at a density of 1 × 10^6^ cells/well and incubated for 24 h. Then, cells were treated with various concentrations (0, 100, 200, and 400 μg/ml) of CME for 24 h, and then annexin V and PI solution were added. Cells were analyzed by flow cytometry (FC500, Beckman).

### 2.5 Fluorescence detection of apoptosis

To detect apoptosis, cells were treated with CME in various of concentration, and annexin V‐FITC (ANXA5‐FITC) and PI staining are conducted according to manufacture protocol. Thus, the morphological manifestation of bubbling cell and ANXA5‐FITC‐ and PI‐stained cells were analyzed under fluorescence microscope (Leica, MDi).

### 2.6 ROS assay

Intracellular ROS generation was analyzed using the ROS Assay Kit (Beyotime) according to the manufacturer's instructions. A549 cells were seeded in 96‐well plates at a density of 1 × 10^4^ cells/well and incubated for 24 h. Then, cells were treated with various concentrations (0, 10, 100, and 200 μg/ml) of CME or 10‐nM taxol for 24 h, and culture media was replaced by dichlorofluorescin diacetate (DCFH‐DA) dilution (1:2000) and incubated for 20 min. Then DCF (oxidative conversion of cell permeable DCFH‐DA to fluorescent DCF) fluorescence distribution was observed by fluorescence microscope (Leica, MDi) at an excitation wavelength of 488 nm and at an emission wavelength of 535 nm.

### 2.7 LDH releases assay

Lactate dehydrogenase (LDH) Assay Kit (Nanjingjiancheng) was employed to assay the effect of CME on release of lactate dehydrogenase in A549 cell line as previously described. Briefly, 549 cells were seeded in 96‐well plates at a density of 1 × 10^4^ cells/well and incubated for 24 h. Then, cells were treated with various concentrations (0, 1, 10, 100, and 200 μg/ml) of CME for 24 h. Then 20 μl supernatants were transferred to a new 96‐well plate and processed following the manufacturer's instructions, and then the absorbance was immediately measured at 450 nm by Microplate reader.

The content of LDH release of 20‐μl supernatants was reflected by activity of LDH calculated according to the following equation:

$$\text{LDH concentration}\;\left(U/L\right)=\frac{\left(\text{Mean OD of test sample}‐\text{Mean OD of the control}\right)}{\left(\text{Mean OD of the standard}‐\text{Mean OD of the blank}\right)}\times \text{standard concentration}\;\left(0.2\;\mu \text{mol}/\text{ml}\right).$$

### 2.8 Caspase‐1 and caspase‐3 activity measurement

A549 cells were plated in 6‐cm^2^ cell culture dishes at a density of 2 × 10^5^ cells/ml and incubated for 24 h and then treated with 200 μg/ml of CME for 2, 8, 12, and 24 h. After that, the cells were washed three times with phosphate buffer saline (PBS) and lysed with 100‐μl radioimmunoprecipitation assay (RIPA) buffer at 4℃ for 30 min. The cell lysates were collected and centrifuged at 12,000 *g* at 4℃ for 10 min. Supernatants were transferred to precooled centrifuge tubes for immediate measurement of caspase‐1, caspase‐3 activities by using caspase‐1, caspase‐3 activity assay kits (Beyotime) according to the manufacturer's protocols. The protein content was measured by bicinchoninic acid (BCA) protein assay kit (Thermo).

### 2.9 Hoechest 33342 staining assay

The culture medium of CME‐treated cells was replaced by fresh culture containing Hoechest 33342 Green Detection Reagent according to the manufacturer's protocol (Ribobio Scientific).

### 2.10 Western blot analysis

Western blot assays were performed as previously described with modification. Briefly, A549 cells were treated with CME (1, 10, 100, and 200 μg/ml) for 24 h. Protein from cell lysates was measured using the BCA assay, separated by electrophoresis, and transferred onto polyvinylidene fluoride (PVDF) membrane (0.45 μm; EMD Millipore). The membrane was blocked with 5% skim milk and washed with tris buffered saline tween (TBST) (0.5% Tween‐50) and incubated overnight with primary antibodies at 4°C. Primary antibodies against cleaved‐caspase‐1 (1:1000), caspase‐3 (1:1000), gasdermin‐D (GSDMD) (1:2000), gasdermin‐E (GSDME) (1:2000), IL‐1β (1:1000), NLRP3 (1:1000), P2X7 (1:1000), TET2 (1:1000), p‐JAK1 (1:1000), phosphorylated‐signal transduction and activators of transcription 3 (p‐stat3) (1:2000), protein 53 (p53) (1:1000), protein 21 (p21) (1:2000), cell division cycle 25B (Cdc25B) (1:1000), cyclinB1 (1:1000), cyclin‐dependent kinase 1 (CDK1) (1:10,000), bcl‐2 (1:5000), BCL2 associated agonist of cell death (BAD) (1:1000), poly‐ADP‐ribose polymerase 1 (PARP1) (1:1000), and α‐tubulin (1:2000) were purchased from Abcam. Membranes were washed three times for 5 min with TBST before addition of goat antimouse or antirabbit horseradish peroxidase‐conjugated secondary antibody (1:5000 dilution, Abcam). The antibody–antigen complexes were visualized by means of enhanced chemiluminescence. The exposure was performed with 5220 Multi (Tanon), and the acquired image was analyzed with Tanon Gis (Tanon).

### 2.11 Analysis of chemical constituents from *C. militaris* by HPLC and ultraperformance liquid chromatography‐quadrupole/time‐of‐flight mass spectrometry

*Cordyceps militaris* extract is solved in double distilled water and centrifugated in 12400 *g*, and supernatant is analyzed by HPLC and ultraperformance liquid chromatography‐quadrupole/time‐of‐flight (UPLC‐Q‐TOF).

High‐performance liquid chromatography assay was carried out on a Waters SunFire™ C18 column (150 mm × 4.6 mm i.d.; 5 μm) with an SGE protecol C18 guard column (250 mm × 4.6 mm, 5 μm) at 35°C. The mobile phase was a mixture of aqueous 0.1% formic acid (eluent A) and pure methanol (eluent B). The gradient elution program was as follows: 0–4 min, 40% B; 5–14 min, 100% B; 15–25 min, 95% B; and 26–30 min, 40% B. The total run time was 30 min, the flow rate was set at 1 ml/min, and the sample volume was 20 μl. The detection wave was 260 nm.

UPLC/ESI‐Q‐TOF‐MS/MS analysis was performed on an Acquity BEH C18 column (100 × 2.1 mm, 1.7 μm, Waters). The optimal mobile phase consisted of a linear gradient system of (A) 0.1% formic acid in water and (B) acetonitrile: 0–0.5 min, 10% B; 0.5–3 min, 10%–20% B; 3–7.5 min, 20%–35% B; 7.5–15 min, 35%–95% B; 15–18 min, 95% B; 18–18.1 min, 95%–10% B; and 18.1–23 min, 10% B. The column temperature was maintained at 30℃. The flow rate was 0.25 ml/min, and the injection volume was 1.0 μl.

### 2.12 Mass spectrometry

Chromatograms were acquired using ESI in both positive and negative ionization mode. The instrumental settings of Q‐TOF‐MS/MS were as follows: Ion source gas 1 and gas 2 were both 55 psi, curtain gas was 35 psi, ion source temperature was 500℃, an ion‐spray voltage of +5500/−4500 V, an declustering potential voltage of 100/−80 V, and a collision energy of ±35 V, and collision energy spread was 15 V. Nitrogen was used as nebulizer and auxiliary gas. Samples were analyzed in both positive and negative ionization modes with scanning mass‐to‐charge (m/z) range from 100 to 1500. Data were collected in information‐dependent acquisition (IDA) mode. Chemical identifications were based on reference standards, chromatographic elution behaviors, chemical composition, mass fragment patterns, and mass spectral library (Natural Products HR MS/MS Spectral Library, Version 1.0, AB Sciex).

### 2.13 Statistical analysis

Data were expressed as mean ± *SD* in excel software. The difference among several means was analyzed by using one‐way Analysis of Variance (LSD or Dannet's T3) in SPSS 20.0 software. *p* < .05 was considered to be statistically significant.

## 3 RESULTS

### 3.1 Cytotoxic effect of CME toward A549

**FIGURE 1:**
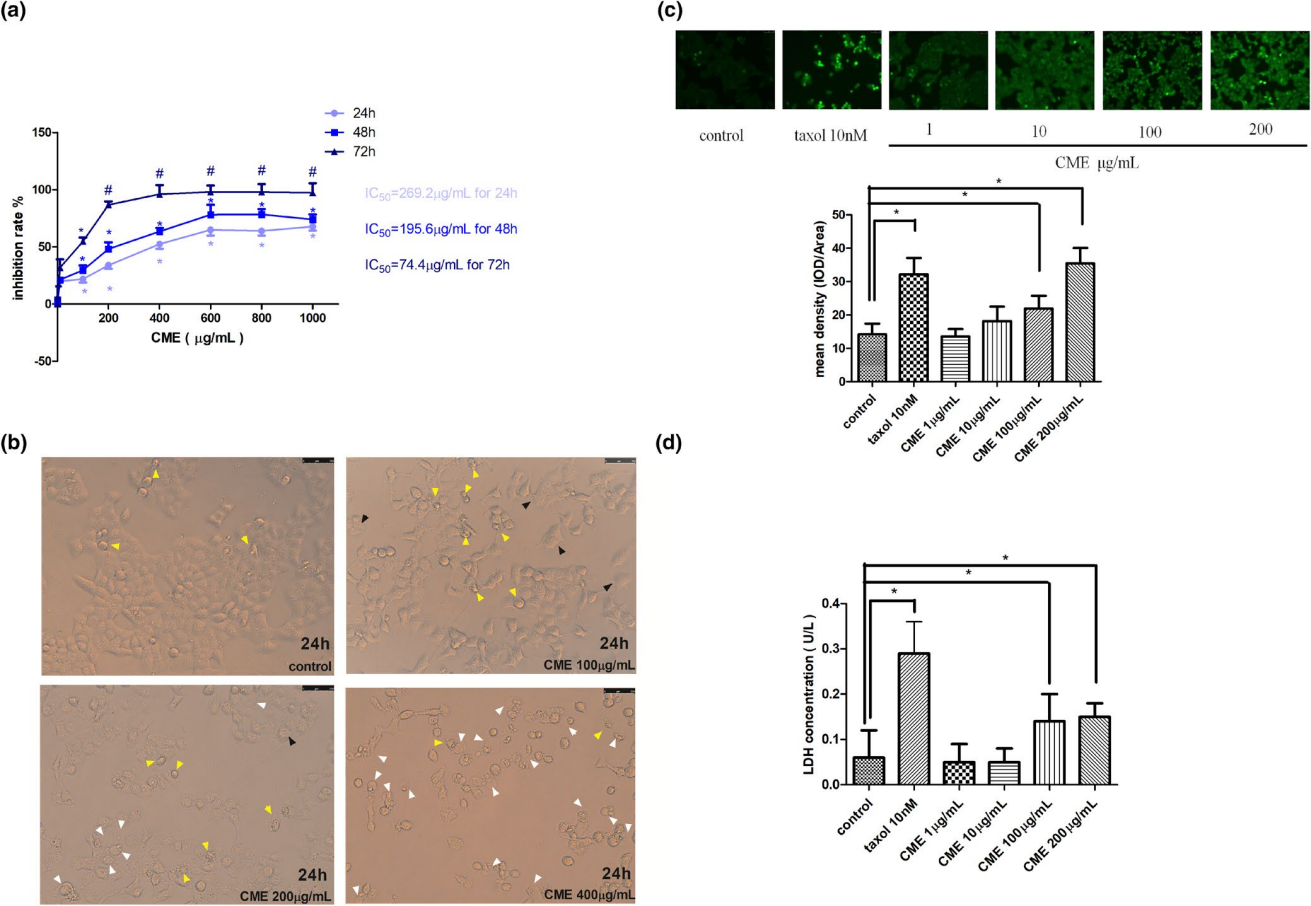
Effect of *Cordyceps militaris* extract (CME) on the viability of A549 cells and observation on cytotoxicity. (a) Inhibitory effect of various concentration of CME on A549 in 24, 48, and 72 h. (b) Morphological observation on cytotoxicity of CME in 24 h. Yellow arrow heads indicate early apoptotic cells with irregular shrinkage; black arrow heads indicate early pyroptosis with cells swell; and white arrows indicate pyroptotic cells with large bubbles blowing form the cellular membrane. (c) Effect of CME treatment on reactive oxygen species generation in A549 cells. (d) Effect of CME on lactate dehydrogenase release in A549 cells. The data represent the mean ± *SD* of six replicates and three independent experiments. *Significant difference from the control (*p* < .05), **Very significant difference from control (*p* < .01)

As Figure 1a shows, the growth of A549 cell was inhibited by CME in a dose and time dependent within concentration of 1–600 μg/ml from 24 to 72 h. At exposure of 600 μg/ml of CME, the inhibition rates are 64.84 ± 1.66% in 24 h and 97.98 ± 1.98% in 72 h. By contrast, growth of A549 is only inhibited by CME in time‐dependent manner from 600 to 1000 μg/ml, and the inhibitory rate are 65.46 ± 1.96% for 24 h, 76.82 ± 2.58% for 48 h, and 97.72 ± 0.34% for 72 h. However, inhibitory effect is not affected by concentration in this section (600–1000 μg/ml). As Figure 1b shows, after 24‐h exposure of CME, cells swell (black arrow), small amount of large bubbles blowing form the cellular membrane (white arrow), and increasing amount of large bubbles are observed in 100, 200, and 400 μg/ml CME treatment. By contrast, number of shrinking cell with irregular membrane (yellow arrow), which is indicated as early apoptosis, is gradually reduced by CME in concentration‐dependent manner.

Compared with the control group, fluorescent DCF, which is converted by ROS from DCFH‐DA, was increased by CME (1–200 μg/ml, 24 h) in a concentration‐dependent manner. Mean density of fluorescent DCF is quantized by Image Pro Plus software, and mean density of ROS index is significantly increased by treatment of CME in 100 and 200 μg/ml (Figure 1c).

A dose‐dependent LDH release was observed in A549 treated with CME (1–200 μg/ml) (Figure 1d). Noticeably, compared with intact cells (0.06 ± 0.01 U/L), CME‐treated cells released more LDH of 0.12 ± 0.02 U/L when they were exposed to CME of 200 μg/ml (*p* < .05).

### 3.2 CME inhibits proliferation of A549 cells and cell cycle proteins

**FIGURE 2:**
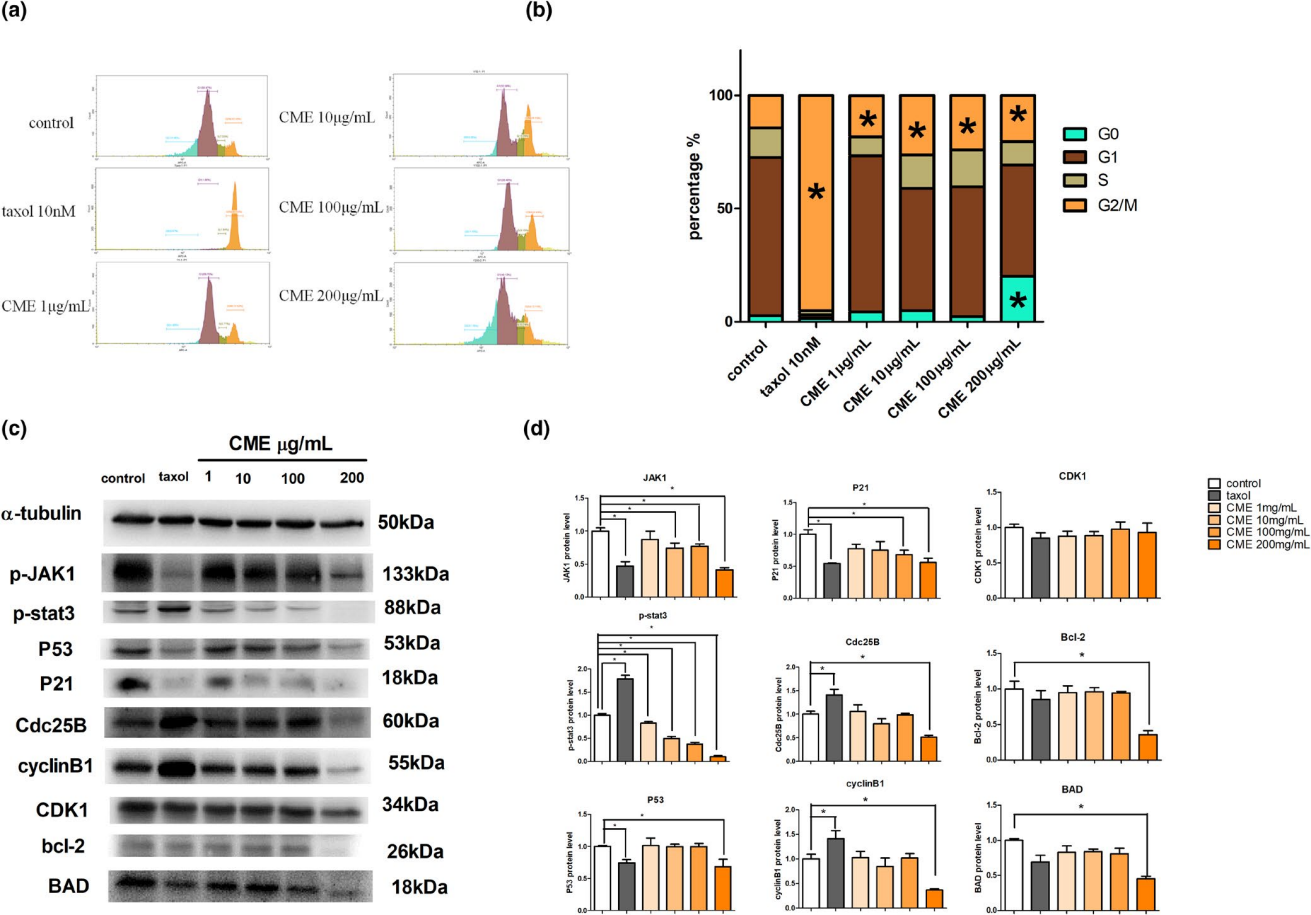
Effect of *Cordyceps militaris* extract (CME) on cell cycle and cycle regulative proteins. (a, b) Graphic representation of cell cycle distribution, data points are the percentage of cells in G0, G1, S, and G2/M at 24 h after treatment. (c, d) Western blotting analysis of lysates (20 µg) prepared from A549 cells treated with taxol or CME at the indicated concentrations for 24 h. Membranes were incubated with antibodies against p‐JAK1, p‐stat3, P53, P21, Cdc25B, cyclinB1, CDK1, and bcl‐2 BAD (protein loading control). The data represent the mean ± *SD* of six replicates and three independent experiments. *Significant difference from the control (*p* < .05)

As Figure 2a,b shows, approximately 19%–28% of A549 cells accumulated in G2/M cell cycle after CME treatment of 1 to 200 μg/ml when compared with control group. Noticeably, approximately 19% of A549 cells accumulated in G0 stage after exposure of CME of 200 μg/ml. Afterward, we investigated the levels of G2/M cell cycle checkpoint proteins in CME‐treated cells by western blotting (Figure 2c,d), which shows that the p‐JAK1, p‐stat3, Cdc25B, cyclinB1, CDK1, Bcl‐2, and BAD protein expressions were significantly inhibited in the concentration of 200 μg/ml while they are hardly affected in lower concentrations.

### 3.3 CME induces apoptosis and pyroptosis‐like cell lysis in A549 cells

**FIGURE 3:**
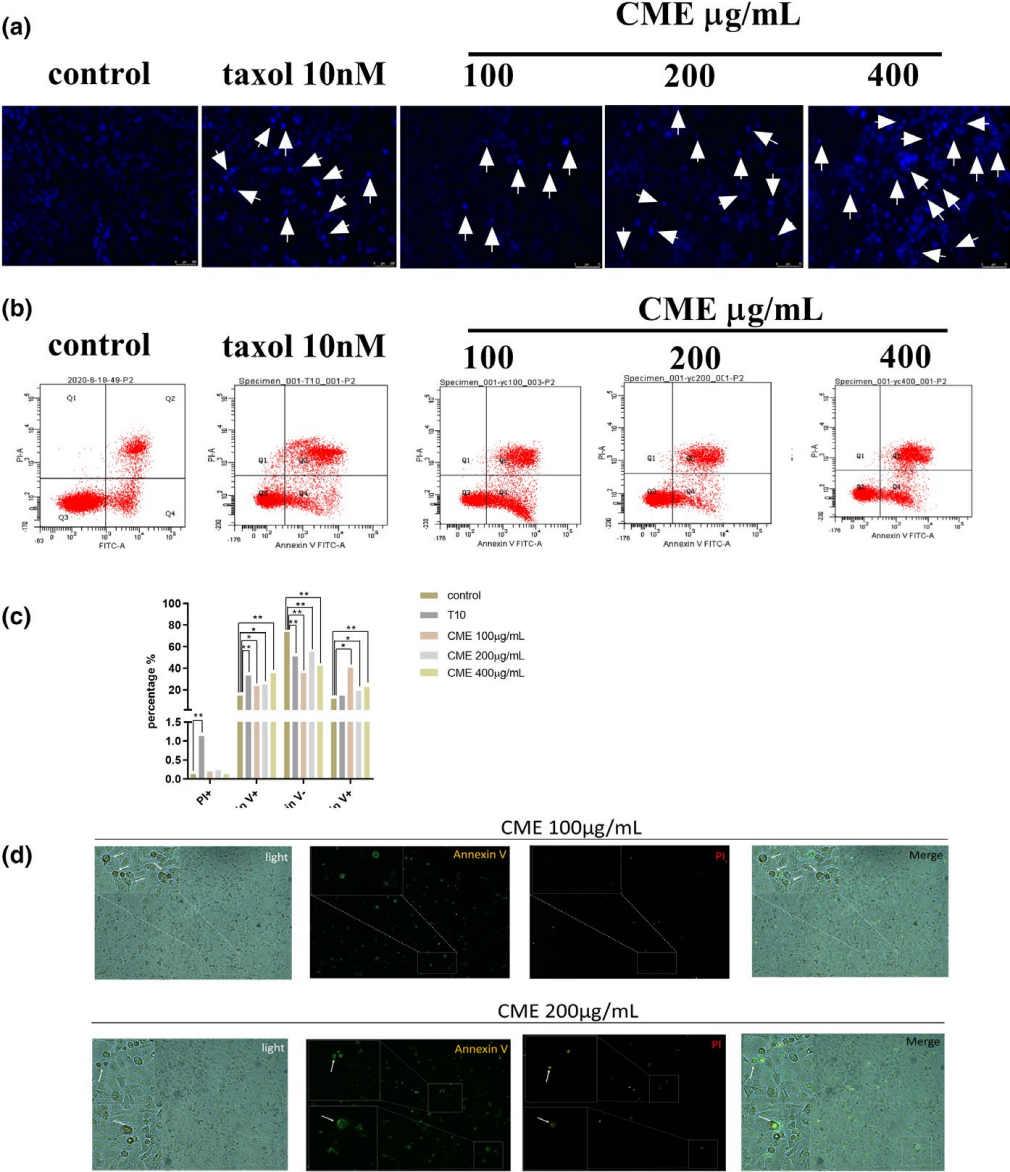
Effect of *Cordyceps militaris* extract (CME) on cell apoptosis. (a) Fluorescence images in A549 cells treated with CME in the different concentration from 100 to 400 μg/ml for 24 h. Cells were stained with Hoechst 33258 (blue) to highlight the nuclei. (b) Flow cytometric analysis of ANXA5‐FITC and PI staining for the determination of apoptosis in A549 cells after treatment of CME for 24 h. (c) Graphic representation of cell apoptosis, data points are the percentage of cells apoptosis at 24 h after treatment. (d) Fluorescence detection of apoptosis in A549 cells after treatment of CME in the concentration of 100 and 200 μg/ml for 24 h. The data represent the mean ± *SD* of six replicates and three independent experiments. *Significant difference from the control (*p* < .05), **Very significant difference from control (*p* < .01)

We examined whether CME induced apoptosis in A549 cells. As Figure 3a shows, amount of condensing point, which represented DNA damage, was increased notably in a dose‐dependent manner after treatment with CME when compared with control. The results of flow cytometry (Figure 3b) confirmed that the percentage of advanced apoptosis (PI+/annexin V+) and the percentages of apoptotic cell death (PI−/annexin V+) were increased by CME treatment in concentration‐dependent manner, especially in the concentration of 100 μg/ml (Figure 3c). As Figure 3d shows, the morphology manifestation of A549 cells bubbling was significantly observed in cells treating with CME of 100 and 200 μg/ml; meanwhile, the annexin V‐FITC (green) and PI (red) staining images show that the bubbling cells with dual positive staining (annexin V^+^ and PI^+^) were only found in the concentration of 200 μg/ml, and annexin V positive cell bubbling was detected in the concentration of 100 μg/ml.

### 3.4 Effect of CME on expression of pyroptosis and apoptosis proteins in A549 cells

**FIGURE 4:**
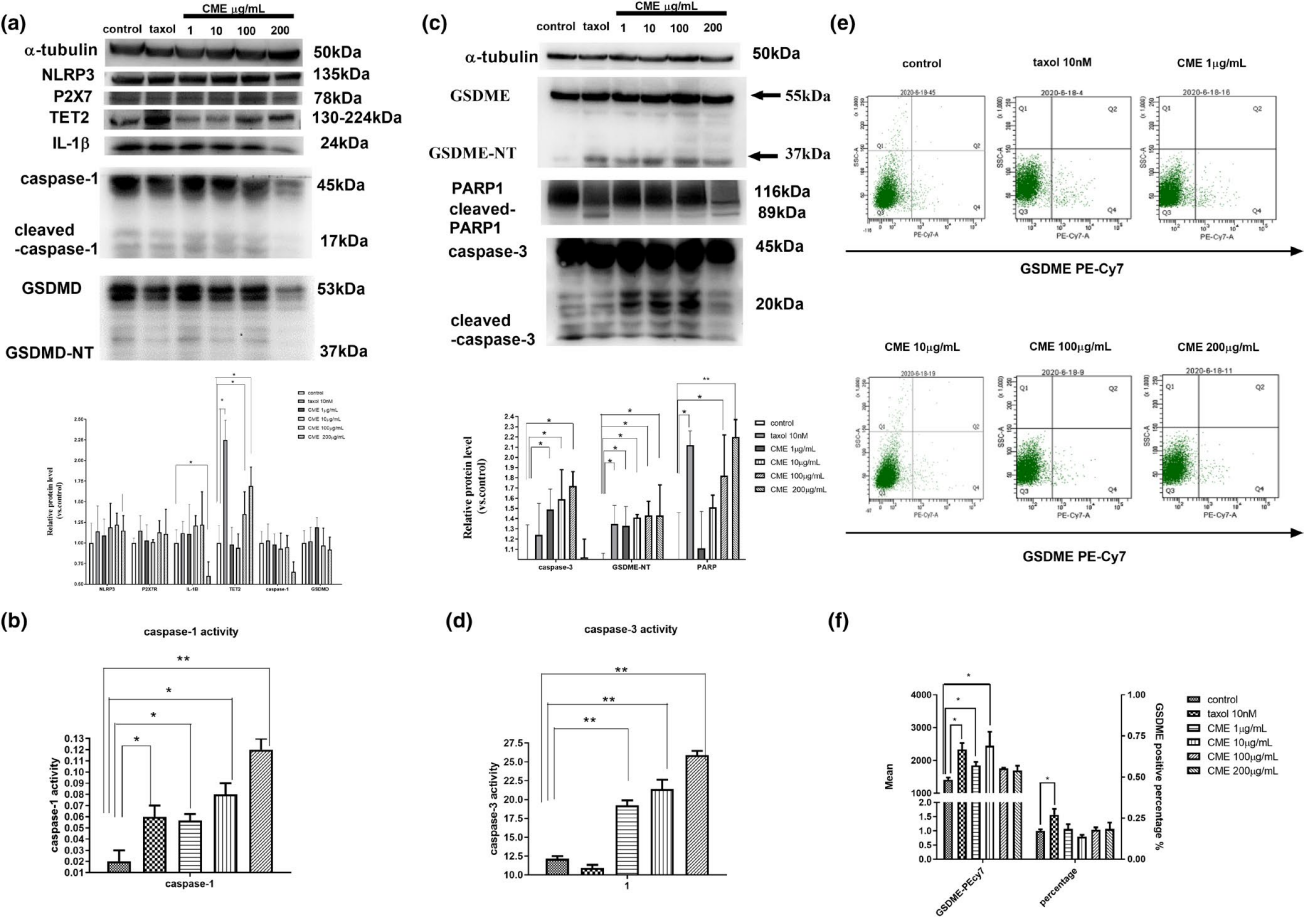
Effect of *Cordyceps militaris* extract (CME) on cells pyroptosis and the caspase‐3/gasdermin‐E (GSDME) pathway in A549 cell line. (a, c) Western blotting analysis of lysates (60 µg) prepared from A549 cells treated with CME or taxol at the indicated concentrations for 24 h. (b, d) Graphic representation of results from the activity of caspase‐1 and caspase‐3 in each experimental group (2, 8, 12, and 24 h). (e) Flow cytometric analysis of GSDME in A549 cells after treatment of CME in different concentration for 24 h. (f) Graphic representation of the positive percentage and mean of GSDME in A549. The data represent the mean ± *SD* of three replicates and three independent experiments. *Significant difference from the control (*p* < .05), ^#^Very significant difference from control (*p* < .01)

As Figure 4a,c shows, protein levels of GSDME, P2X7R, NLRP3, IL‐1β, cleaved‐caspase‐3, caspase‐1, and cleaved‐PARP were significantly upregulated by CME in concentration‐dependent manner. Meanwhile, as Figure 4b,d shows, the activity of caspase‐1 and caspase‐3 was progressively promoted in time‐dependent manner. Moreover, as Figure 4e,f showed, mean potency of PE‐Cy7, which represents overall membrane expression of GSDME, was upregulated by CME, especially in the concentration of 10 μg/ml. By contrast, membrane expression of GSDME is slightly affected in 100 and 200 μg/ml.

### 3.5 Effect of CME on viability and morphology was revered by caspase‐3 inhibitor Ac‐DC

**FIGURE 5:**
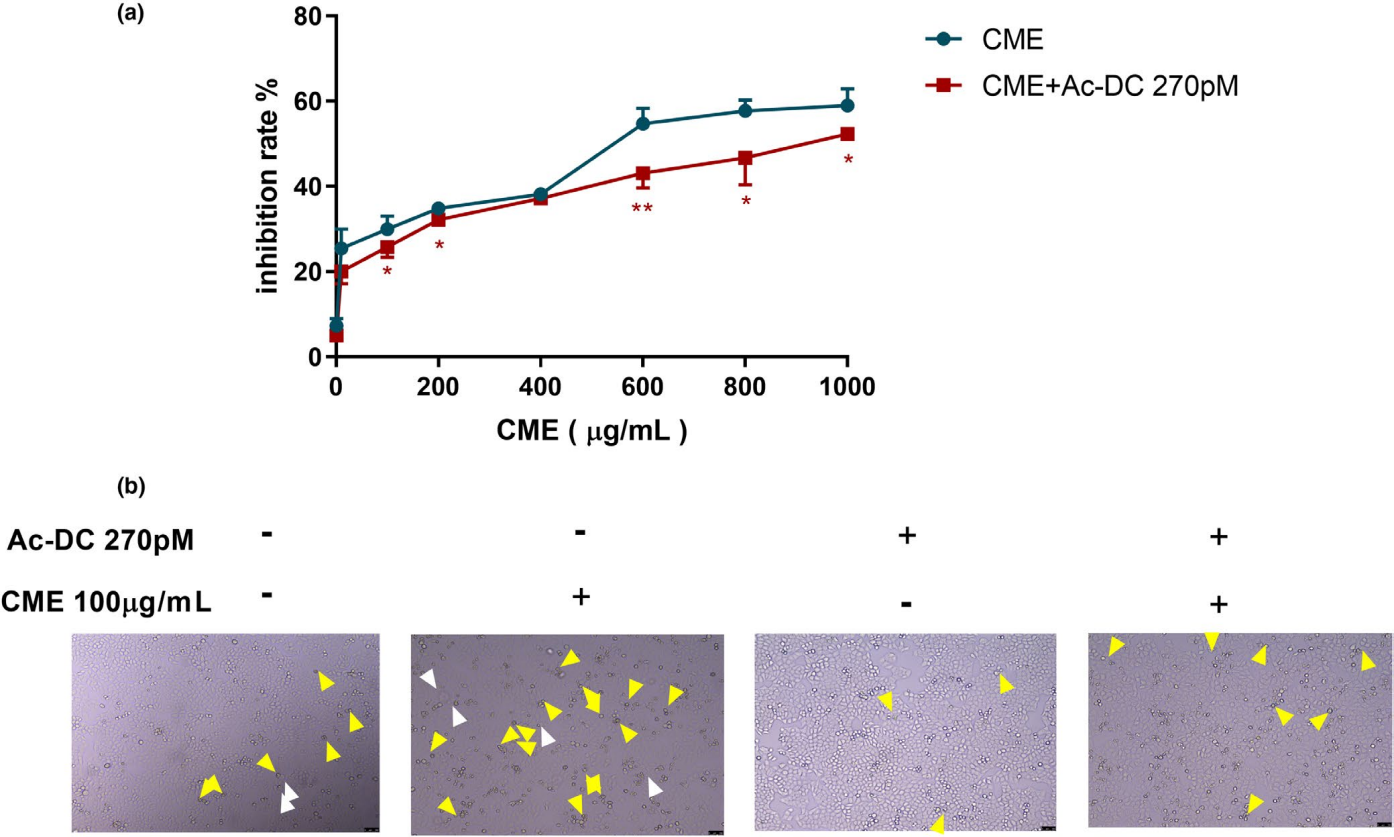
Effect of *Cordyceps militaris* extract (CME) on viability and morphology in the present of caspase‐3 inhibitor Ac‐DEVD‐CHO (Ac‐DC). (a) Inhibitory effect of various concentration of CME on A549. Data points are the percentage (%; AC‐Dc 270pM treated/AC‐Dc 270pM untreated) relative to untreated cells at same time point. (b) Morphological observation on cells pyroptosis. (Yellow arrow heads indicate early apoptotic cells with irregular shrinkage, and white arrows indicate pyroptotic cells with large bubbles blowing form the cellular membrane.) The data represent the mean ± *SD* of six replicates and three independent experiments. *Significant difference from the control (*p* < .05), **Very significant difference from control (*p* < .01)

As Figure 5a shows, inhibition rate is elevated along with uphill of the CME concentration (1–1000 μg/ml). On the contrast, inhibitory effect of CME is reversed in present of AC‐Dc 270pM, and this reversion is more significant in the high concentration of CME (600 and 800 μg/ml). As Figure 5b shows, after 24‐h exposure of CME of 100 μg/ml, major amount of shrinking cell with irregular membrane (yellow arrow) and minor ballooning cells (white arrow) remarkably increased. Meanwhile, amount of shrinking cell and bubbling cells were reduced by CME combining AC‐Dc of 270pM.

### 3.6 Inhibitory effect of CME on pyroptosis and apoptosis was reversed by capase‐3 inhibitor Ac‐DC

**FIGURE 6:**
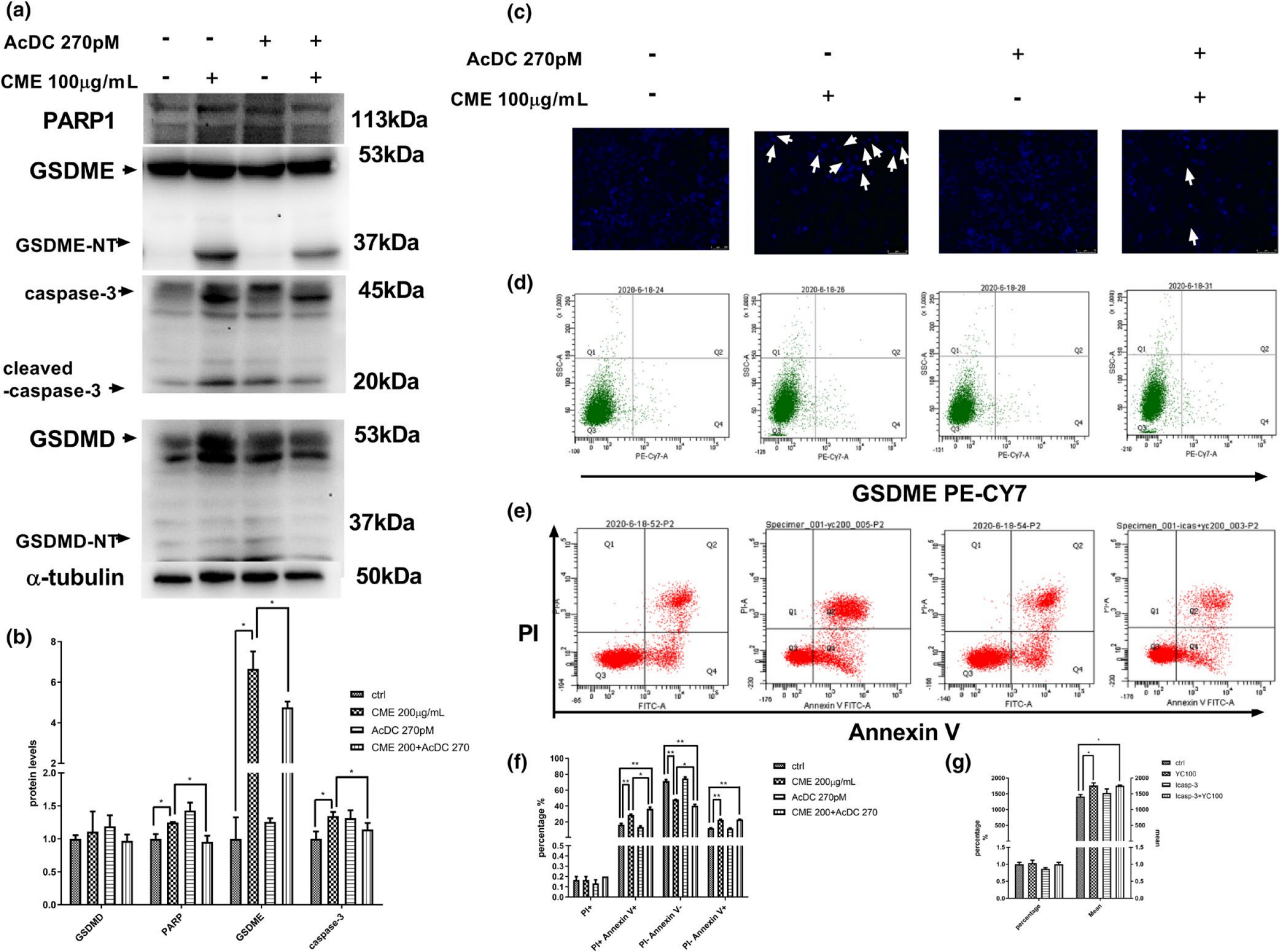
Effect of *Cordyceps militaris* extract (CME) on caspase‐3/gasdermin‐E (GSDME) pathway and cell morphology in present of Ac‐DEVD‐CHO (Ac‐DC). (a) Western blotting analysis of lysates (20 µg) prepared from A549 cells treated with CME 100 μg/ml or AC‐Dc 270pM for 24 h. (b) Graphic representation of result from protein levels of A549 cell after treatment of CME 200 μg/ml or AC‐Dc 270pM for 24 h, Data points are gasdermin‐D (GSDMD), poly‐ADP‐ribose polymerase (PARP), GSDME, and caspase‐3. (c) Fluorescence images in A549 cells treated with CME 100 μg/ml or AD‐Cd 270pM for 24 h. Cells were stained with Hoechst 33258 (blue) to highlight the nuclei. (d) Flow cytometric analysis of GSDME in A549 cells after treatment of CME in different concentration for 24 h. (e) Flow cytometric analysis of ANXA5‐FITC and PI staining for the determination of apoptosis in A549 cells after treatment of CME for 24 h. (f) Graphic representation of GSDME PE CY‐7 and SSC staining for the determination of cell pyroptotic in A549 cells after treatment of CME 200 μg/ml or AC‐Dc 270pM for 24 h, data points are the (PI+/annexin V+) or (PI−/annexin V+). (g) Graphic representation of the percentage and mean of GSDME in A549, data points are the CME 100 or Ac‐DC at 24 h after treatment. The data represent the mean ± *SD* of six replicates and three independent experiments. *Significant difference from the control (*p* < .05), **Very significant difference from control (*p* < .01)

In experiment of western blotting (Figure 6a,b), apoptosis‐related proteins, such as cleaved‐caspase‐3 and pyroptosis‐related protein GSDME‐NT, were increased in CME‐treated A549 cells, whereas they were downregulated in the presence of Ac‐DC 270pM, a specific caspase‐3 inhibitor. It was observed that CME remarkably enhanced the expression of the protein levels of GSDME and cleaved‐caspase‐3, and the treatment of CME with Ac‐DC 270pM inhibited their expression. Correspondingly, as Figure 6c showed, condensing point of Hoechst 33258 (blue) appeared after 24‐h exposure of CME, and CME‐induced DNA condensing was reduced in present of Ac‐DC. To sum up, this phenomenon confirmed that pyroptosis was caspase‐3 dependent. The similar trends were found in flow cytometry of ANXA5‐FITC and PI staining, as well as GSDME PE CY‐7 staining of treated cells. CME‐induced apoptosis and pyroptosis were downstream of caspase‐3, and they may share some phenomena (annexin V staining and DNA damage) as shown in Figure 6d–g.

### 3.7 Constituent analysis of CME by HPLC and UPLC‐TOF

**FIGURE 7:**
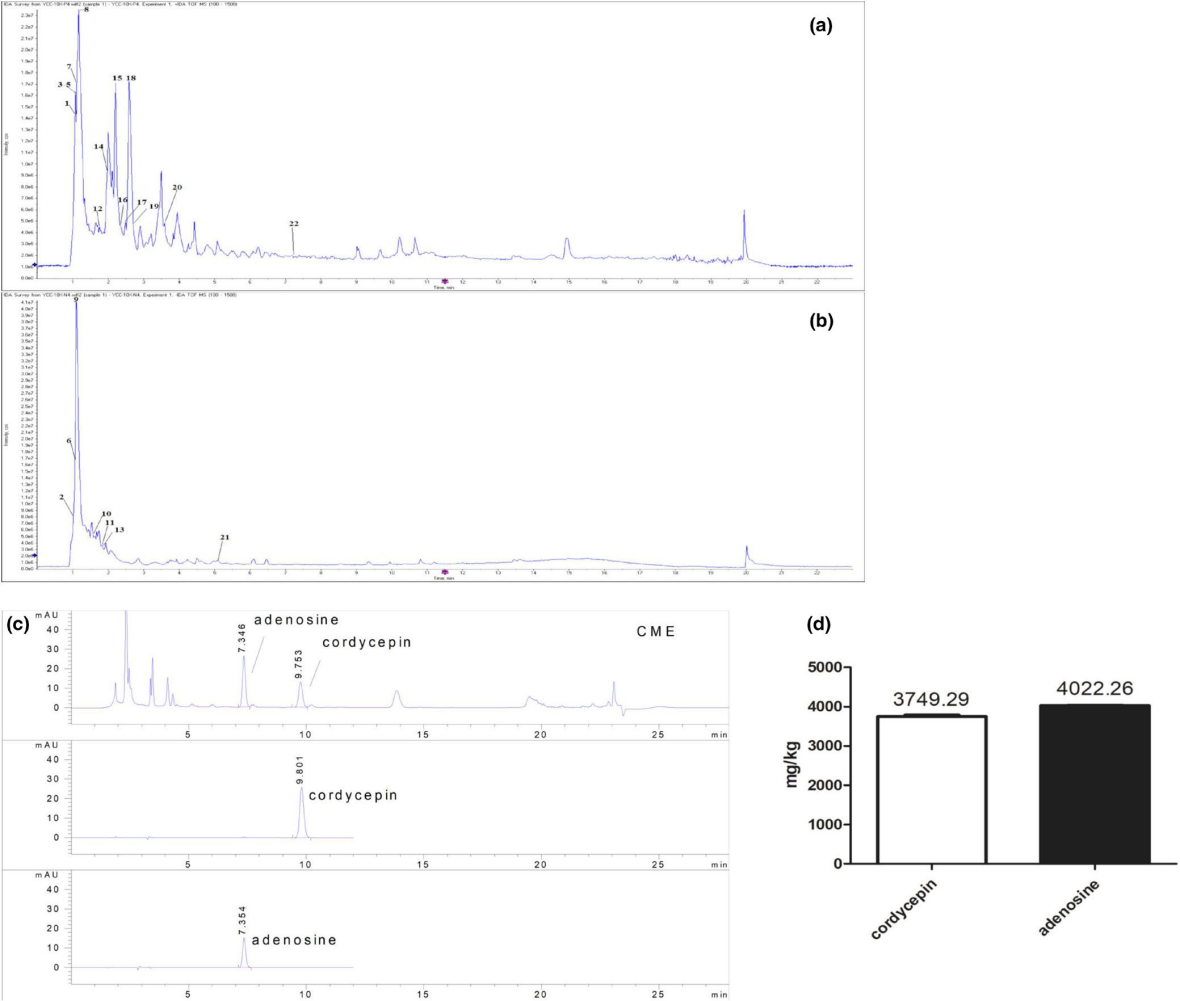
Chemical component analysis and qualification of bioactive component in *Cordyceps militaris* extract. Total ion chromatograms of ultraperformance liquid chromatography‐quadrupole/time‐of‐flight mass spectrometry by positive mode (a) and negative mode (b) for extraction of *C. militaris*. (c) High‐performance liquid chromatograph identification of cordycepin and adenosine in *C. militaris*. (d) Contents of cordycepin and adenosine in *C. militaris* extract

According to the research of chemical constituents in *C. militaris*, nucleotides and nucleotide derivatives, including cordycepin, were effective components in Cordyceps. Twenty‐two small molecules of CME were identified by UPLC‐Q‐TOF (Figure 7a,b), and they were further classified by biofunction and contribution to cancer treatment through reviewing academic articles (Table 1). They were composed of trophism, neoadjuvant component, cytotoxic component, and cancer deterioration promoter as described as follows.

**TABLE 1:** Identification of chemical constituents in *Cordyceps militaris* by ultraperformance liquid chromatography‐quadrupole/time‐of‐flight mass spectrometry

No.	Rt (min)	Formula	Ion mode	Accurate mass (*m*/*z*)	△ppm = accurate mass − theoretical mass	Fragment ions (*m*/*z*)	Identification	Anticancer effect or related biofunction
1	1.02	C_6_H_14_N_2_O_2_	[M+H]^+^	147.1131	2.0	130.0871, 84.0809	Lysine	Amino acid as nutrition
2	1.03	C_6_H_9_N_3_O_2_	[M−H]^−^	154.0623	0.9	137.0356, 93.0456	Histidine	Amino acid as nutrition
3	1.05	C_5_H_9_NO_2_	[M+H]^+^	116.0706	0.3	70.0651	Proline	Amino acid as nutrition
4	1.07	C_9_H_9_N_5_O_3_	[M+H]^+^	236.0788	4.2	192.0933, 177.0676, 162.0445	6‐Succinoaminopurine	No related report is found
5	1.08	C_6_H_14_N_4_O_2_	[M+H]^+^	175.1184	−2.9	158.0933, 130.0980, 116.0707	Arginine	Amino acid as nutrition
6	1.10	C_6_H_14_O_6_	[M−H]^−^	181.0719	0.6	163.0615, 89.0248	Mannitol	Nephrotoxicity prevention in chemotherapy (Makimoto et al., 2018; McKibbin et al., 2016)
7	1.13	C_5_H_9_NO_4_	[M+H]^+^	148.0609	2.9	102.0562, 84.0444	Glutamic acid	Amino acid as nutrition
8	1.14	C_5_H_11_NO_2_	[M+H]^+^	118.0861	−1.2	58.0649	Betaine	Oxidative stress‐mediated apoptosis and inflammation inducer (Yang et al., 2019); risky factor for lung cancer (Swartz et al., 2013)
9	1.15	C_12_H_22_O_11_	[M−H]^−^	341.1090	0.2	179.0554, 161.0472	Trehalose	Candidate apoptosis inducer (Ichihara et al., 2017)
10	1.59	C_9_H_12_N_2_O_6_	[M−H]^−^	243.0623	0.2	200.0612, 110.0243	Uridine	Source for mediating cancer epithelial to mesenchymal transition (Finley, 2019)
11	1.74	C_10_H_13_N_5_O_5_	[M−H]^−^	282.0846	0.7	150.0423	Guanosine	Potential factor for carcinoma cell growth (Huang, Ni, et al., 2018)
12	1.75	C_6_H_5_NO_2_	[M++H]^+^	124.0395	1.6	78.0347	Nicotinic acid	In vitro cytotoxic through reactive oxygen species generation pathway (Lehmler et al., 2008)
13	1.79	C_4_H_6_O_4_	[M−H]^−^	117.0193	−0.2	73.0294	Succinic acid	No cytotoxic is reported
14	1.92	C_6_H_6_N_2_O	[M+H]^+^	123.0548	−3.6	106.0300, 80.0498, 78.0343	Nicotinamide	Source for cell proliferation in A549 cell line (Sartini et al., 2015)
15	2.19	C_9_H_11_NO_3_	[M+H]^+^	182.0816	2.4	136.0759, 119.0495	Tyrosine	Amino acid as nutrition
16	2.28	C_10_H_13_N_5_O_4_	[M+H]^+^	268.1040	0	136.0761, 119.0503	Adenosine	Induce A549 cell apoptosis of 0.01–10 mM (Kamiya et al., 2012) and senescence (Yang et al., 2013)
17	2.42	C_6_H_13_NO_2_	[M+H]^+^	132.1019	0	86.0970	Leucine	Amino acid as nutrition
18	2.60	C_10_H_13_N_5_O_3_	[M+H]^+^	252.1082	−3.6	136.0617, 119.0349	Cordycepin	Stimulation of the cell apoptosis and the cell cycle arrest via caspases activation in chemotherapy‐resisted cancer (Cho & Kang, 2018), inhibit Nuclear Factor kappa B pathway in A549 (Zhang et al., 2015), and inhibit NO pathway (Hwang et al., 2017)
19	2.61	C_5_H_5_N_5_	[M+H]^+^	136.0614	−2.4	119.0350, 92.0246	Adenine	Metabolites in NAD pathway
20	3.58	C_9_H_11_NO_2_	[M+H]^+^	166.0866	1.9	120.0808, 103.0545	Phenylalanine	Amino acid as nutrition
21	5.08	C_11_H_12_N_2_O_2_	[M−H]^−^	203.0820	−3.2	159.0959, 116.0507	Tryptophan	Amino acid as nutrition
22	7.12	C_17_H_20_N_4_O_6_	[M+H]^+^	377.1463	1.9	243.0893, 172.0886	Vitamin B_2_	Potential factor for preventing carcinogenesis (Bassett et al., 2012)

#### 3.7.1 Trophism

They served as nutritional component, such as lysine, histidine, proline, arginine, glutamic acid, tyrosine, leucine, phenylalanine, and tryptophan.

#### 3.7.2 Neoadjuvant component

Some of chemical constituents were reported to protect cell and organ during chemotherapy in some published articles, for example, the mannitol was detected to a nephrotoxicity prevention in chemotherapy, and the vitamin B2 was found to have a potential factor for preventing carcinogenesis.

#### 3.7.3 Cytotoxic component

Chemical constituents such as betaine, trehalose, nicotinic acid, adenosine, and cordycepin had the cytotoxic effect toward cancer. They induced cell apoptosis and inhibit proliferation according to previous research.

#### 3.7.4 Cancer deterioration promoter

Some of chemical constituents were found to promote deterioration in reported cancer treatment, such as, betaine, uridine, guanosine, and nicotinamide.

Other chemical constituents have not been reported in term of cancer treatment, like 6‐succinoaminopurine, succinic acid, and adenine.

Components of CME were analyzed by UPLC‐TOF, and representative component is qualified by using HPLC. The results show cordycepin and adenosine are detected in CME, and the contents of them were 3749.29 ± 36.90 and 4022.26 ± 13.58 mg/kg, respectively, in CME (Figure 7c,d).

## 4 DISCUSSION

**FIGURE 8:**
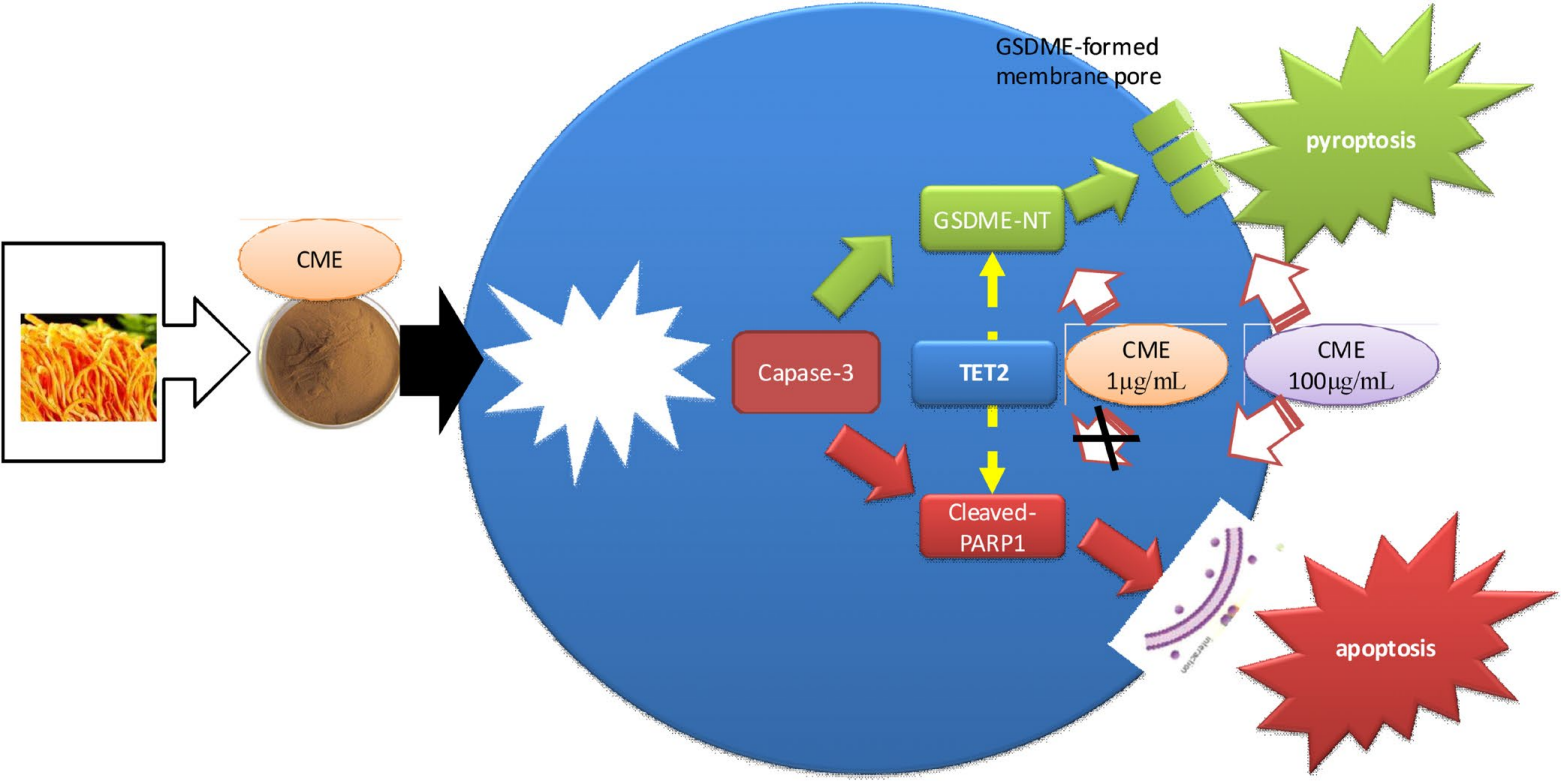
Graphic abstract of *Cordyceps militaris* extract (CME) causing pyroptosis and apoptosis in A549

First‐line anticancer drugs are mainly designed to target specific molecule to cause malignant cell apoptosis, whereas dietary therapy may benefit cancer treatment in an alternative manner. CME, one of well‐known dietary therapy, could serve as a complementary medicine in anticancer treatment. However, it requires more scientific validation on its anticancer effect to facilitate clinic application of CME. In our study, it is validated that CME exerted cytotoxicity toward A549 cell line through inducing caspase‐3‐dependent apoptosis and pyroptosis. To our knowledge, this study firstly reported pyroptosis‐causing effect of CME against A549 cell line in vitro along with regulation on caspase‐3‐dependent GSDME pathway (shown as Figure 8).

### 4.1 CME causes pyroptosis through caspase‐3/GSDME pathway in A549 cell line

To date, increasing attention is paid to pyroptosis in cancer research due to its alternative role in improving efficacy of cancer treatment (Abe & Morrell, 2016). Pyroptosis is one of the novel inflammation‐involved PCD, which depended on ROS/inflammasome/caspase‐1,3 activation (Chen et al., 1996). Furthermore, GSDMD and GSDME are identified as molecular effectors and markers of pyroptosis, and they can be activated to form membrane pores (Sborgi et al., 2016). Damaged membrane results in cell bubbling and subsequent lytic cell death. Additionally, ROS generation, which can be triggered by anticancer agents, acts as secondary messengers for activate inflammasome/caspase‐1/GSDMD and caspase‐3/GSDME pyroptotic pathway (Yang et al., 2016). In our research, it is found that CME exerts antitumor effect toward A549 along with causing cell bubbling, upregulating intracellular ROS, and activating caspase‐3/GSDME pathways, implying that pyroptosis is induced by CME in the putative pathway.

Cell bubbling, which is a morphological feature of pyroptosis, is reported to be induced by chemotherapy or herbal extract in A549 cell line (Liang et al., 2020; Wang et al., 2017). Consistently, cell bubbling is induced by CME in our research. Furthermore, large cell bubbles, which undergo advanced pyroptosis, are also induced by high concentration of CME. Cell bubbling is found to be associated with GSDME activation in previous research (Yu et al., 2019). In our research, both of CME‐induced cell bubbling and GSDME activation are validated to be caspase‐3 dependent, and cleaved‐GSDME can cause cell bubbling through forming membrane pores. Therefore, caspase‐3/GSDME axis is assumed to be responsive for initiating CME‐induced cell bubbling. Moreover, advanced stage of CME‐induced pyroptosis, which is charactered as high expression of GSDME‐NT membrane pore (Lei et al., 2018), is further validated to be dependent of caspase‐3 in our research. Specifically, membrane GSDME‐NT is seldom detected, whereas plasma GSDME‐NT is extensively highlighted in current research. Therefore, flow cytometry method (GSDME antibody conjugated with PE‐cy5 probe) is set up to measure membrane GSDME in our research. As results show, overall membrane GSDME expression is slightly increased, whereas individual expression is remarkably enhanced in specific single cell. These results present differentiated effect of CME on activating GSDME in comparison with outcome in western blotting. Outcome of flow cytometry shows more consistent with change in advanced cell bubbles in morphological observation, and they indicated that GSDME is extensively activated, but few of them are finally formed in membrane. Moreover, in bubbling cells and advanced ones, it is positively stained with membrane damage probe annexin V and even dual positive staining of annexin V and PI. This finding indicates that structural damage of membrane and permeability (detected by intracellular PI) alteration are progressively induced by CME in pyroptotic cell, and this phenomenon may be also attributed to GSDME‐forming membrane pore. However, it is still not exclusive that apoptosis may be stimulated in pyroptotic cell at the same time, and more exploration is needed to make a more specific conclusion. Additionally, GSDME‐induced cell bubbling can further lead to structural damage of cell membrane, named cell lysis. Pyroptotic cell debris is source of immunogenic response and cause immunogenic cell death (Sansone et al., 2021). Thus, CME‐induced cell bubbling or cell lysis may exhibit indirect anticancer effect through pyroptosis‐involved immunity response. However, interaction between immunity cell and pyroptotic cancer cell is not included in our research; therefore, our finding only represents partial effect of CME‐induced pyroptosis toward A549.

Gasdermin‐E is currently reported as an important enzyme for prospectively improving cancer treatment with dual mediations including induction of pyroptosis and immunocyte infiltration (Peng et al., 2020; Zhang et al., 2019). GSDME will be valuable target for refining CME as dietary therapy, and likewise, upstream regulation of GSDME is worth exploring as well. In term of upstream pathway regulating pyroptosis, caspase‐1 and caspase‐3 are responsive enzymes for catalyzing GSDMD and GSDME, respectively (Shalini et al., 2015). Caspase‐1 and caspase‐3 are promoted by CME in a concentration‐ and time‐dependent manner. However, protein expression of cleaved‐GSDMD, which is substrate of caspase‐1, is not affected by CME treatment. By contrast, protein expression of cleaved‐GSDME, which is substrate of caspase‐3, is significantly upregulated by CME. Taken together, our finding confirmed that CME‐induced cell death favored caspase‐3/GSDME‐involved pyroptosis rather than caspase‐1/GSDMD as previous research reported (Zhang et al., 2019). Potential factors interpreting selectivity of caspase‐3 are needed to explain this phenomenon in CME‐induced pyroptosis (Ramirez et al., 2018). Furthermore, caspase‐3/GSDME pyroptosis and caspase‐3/PARP apoptosis are reported to be simultaneously induced by chemotherapy drug in A549 (Zhang et al., 2019). However, in our research, caspase‐3/GSDME can be activated without affecting apoptosis due to our finding that caspase‐3/GSDME is stimulated by CME in 10 μg/ml, which did not show cytotoxic effect toward A549 or regulation on apoptosis markers PARP. It is indicated that caspase‐3/GSDME is selectively activated by CME of 10 μg/ml. However, its mechanism underlying selectivity of caspase‐3/GSDME is not clear so far.

In summary, pyroptosis‐featured cell morphology and ROS/caspase‐3/GSDME pyroptotic pathway are activated by CME. CME‐induced pyroptosis and GSDME regulation are validated to be caspase‐3 dependent in loss‐of‐function assay, which may provide a novel insight for understanding anticancer property of CME.

### 4.2 CME causes caspase‐3/PARP apoptosis and mild cell cycle arresting, but caspase‐3/TET2 regulation is unexpectedly discovered

DNA damage is one of apoptotic markers, and it triggers caspase‐3, which is a protease to conduct PCD (Choudhary et al., 2015). DNA damage will finally lead to apoptotic alternations including membrane structure damage and high permeability, which is characterized as shrinking cells. Breakdown of DNA stems from CME‐induced lipid peroxidation and its oxidative impairment on nucleotide (Bao et al., 2018). In the previous research, CME‐induced apoptosis is correlated with caspase‐3 activation (Lee et al., 2006) and genotoxic effect (Li et al., 2020); however, mechanism is still not validated. It is not convincing enough for clinical doctor to use CME without validated target. By using loss‐of‐function assay, apoptotic cell alternation and genotoxic effect are validated to be partially caspase‐3 dependent in CME‐induced cell death in our findings. Moreover, CME‐induced cell shrinking is reversed in present of caspase‐3 inhibitor, indicating cell shrinking is partially processed through DNA‐damage/caspase‐3 specific pathway. Taken together, it is assumed that CME‐induced ROS impairs chromatin and stimulate intrinsic apoptosis cascade with progressive activation of ion channels and pores, which leads to occurrence of shrinking cell. As a dietary therapy, it is valuable to find this potential target and provide insight into its clinic usage. Specifically, CME‐related potentiation of caspase‐3 may serve as regulative target for further in‐depth investigation or clinic application to some extends. Additionally, caspase‐3‐dependent apoptosis accounts for few percentages in CME‐induced cell death in 24‐h exposure. Therefore, non‐caspase‐3‐dependent apoptosis (Brentnall et al., 2013; Lamkanfi & Kanneganti, 2010) should be further validated in CME‐induced cell death as well.

Poly‐ADP‐ribose polymerase 1, which is a chromatin‐associated enzyme, can be activated by DNA strand breaks and mediates apoptotic cell death (Nargi‐Aizenman et al., 2002). In our research, CME‐lead chromatin damage and cleaved‐PARP1 expression are reversed by caspase‐3 inhibitor Ac‐DC, and assumptions of previous research are validated in this finding. In other words, CME‐induced damaged DNA stimulates caspase‐3 and afterward caspase‐3/PARP, which is followed cascade of apoptosis. More specifically, in term of intracellular translocation of caspase‐3, caspase‐3 is activated to exhibit genotoxicity through cytoplasm‐nuclear migration (Wang et al., 2019). This process can be inhibited by caspase‐3 inhibitor. Therefore, intranuclear caspase‐3/PARP activation is assumed to be suppressed as well. Even though ROS can damage DNA through oxidation, cancer cell still can survive through counteracting this damage by redox homeostasis. Because this homeostasis can be impaired by caspase‐3/PARP axis in term of genotoxicity (Ko et al., 2004), cleaved‐PARP is assumed to be upstream regulator of genotoxic mediation of CME. Therefore, ROS/caspase‐3/PARP pathway can worsen DNA damage instead of repair. However, CME‐induced DNA damage is reversed by caspase‐3 inhibitor. Taken together, DNA damage can be the cause of caspase‐3 activation and vice versa, and this finding indicates that mutual interaction may occur between caspase‐3 and DNA damage under CME exposure. To further explain this phenomenon, this interaction may be mediated by autophagy‐apoptosis homeostasis according to Li Xu's finding (Xu et al., 2016).

Cell cycle arresting is common target of anticancer therapy, and it is slightly affected by CME. However, it reflects antiproliferative effect and proapoptosis effect of CME to some extends. Cell cycle check point proteins cyclinB1, CDC25B, and CDK1 and regulative proteins JAK, P53, and P21 are inhibited only in 200 μg/ml of CME except for phosphoration form of STAT3, which is progressively repressed by CME. Noticeable, p‐STAT3, which is a nuclear protein regulating cell cycle and also a multidrug resistance (MDR) protein resisting apoptosis, is remarkably inhibited. Our results indicated that apoptosis‐inducing effect of CME may be associated with STAT3‐related cell proliferation pathway as previous research (Fathi et al., 2018). Additionally, occurrence of G0 phase, which reflected damage of chromatin (Pietrzak et al., 2018), highlights DNA‐damaging effect of CME in turn.

Unexpectedly, TET2 is upregulated by CME treatment, and it is revered by caspase‐3 inhibitor. Due to methylation regulation of TET2, it may switch transcriptional expression of antiapoptosis or proapoptosis gene in this epigenetic regulation. Taken together, TET2 may play a crucial role in caspase‐3‐involved mechanism in epigenetic manner (Zhaolin et al., 2019), and TET2 is reported to be response for DNA damage (Chen et al., 2018), which may alternatively explain mutual interaction between caspase‐3 and DNA damage. Therefore, validation of TET2‐related effect in CME‐induced pyroptosis is undergone in our lab. Additionally, CME‐caused suppression in STAT3 may play a role in switching cell death from apoptosis to pyroptosis due to abrogating effect of STAT3 on apoptosis through epigenetic regulation (Samanta et al., 2018). Conclusively, our data provided basic insight into role of caspase‐3/TET2 in survival or death switch after CME treatment, and it will be beneficial to define regulating network for pyroptosis and refining CME.

### 4.3 Putative bioactive component based on HPLC‐qTOF and references

As a dietary therapy, CME exerts mild anticancer effect with validated and potential targets, refinement is needed through enriching bioactive component according to our findings. Extraction and efficacy of CME can be refined through screening potential bioactive components (nicotinic acid, betaine, adenosine, and cordycepin) and dismissing unnecessary components (amino acid, some nucleotides, and cancer deterioration promoters). According to previous research (Yang et al., 2019), betaine, trehalose, nicotinic acid, adenosine, and cordycepin were reported to possess the cytotoxic effect toward cancer. Specifically, nicotinic acid is stimuli for generating ROS and exerting cytotoxicity as well (Lehmler et al., 2008), indicating it may be one of responsive component for inducing pyroptosis. It will be beneficial for improving its efficacy if bioactive component is enriched in CME. Moreover, fermentation or bioactive‐enrichment separation is possible refinement method to refine CME based on our findings, and it will advance clinic usage of CME as dietary therapy for cancer treatment.

The limit of this research is that effector of pyroptosis is not investigated and in vivo experiment is not included. Neutrophils or macrophage is required for cell lysis in advanced pyroptosis (Kovacs & Miao, 2017), and they can be recruited by interleukins secreted by pyroptosis cancer cells. Additionally, immunogenicity may be directly induced by some stress inducer in CME (Wang et al., 2017) or indirectly induced by CME‐lead endogenous lipid peroxidatant (Kang et al., 2018) of A549 cell line, but it is not included in our research.

## 5 CONCLUSION

*Cordyceps militaris* extract, which is hydrosoluble fraction of dietary herb for cancer patients, is validated to induce PCD in A549 cell line through concomitantly activating caspase‐3‐dependent caspase‐3/PARP apoptosis and caspase‐3/GSDME pyroptotic pathways in vitro. Additionally, profile investigation of CME component, mild cell cycle arresting, and cell proliferation‐inhibiting effect provide further understanding on the feature of CME's anticancer property. In sum, our finding highlights rationale for clinic application of CME in NSCLC treatment.

## ACKNOWLEDGMENTS

The author would like to acknowledge the supportive work from Zhongshan Zuojian Biological Technology Co. Ltd and all the co‐authors. This work also was supported by National Natural Science Foundation of China (grant number 81202961); Natural Science Foundation of Guangdong Province (grant number 2018A0303130160); Guangzhou Science and Technology Project (grant number 201904010180) and Administration of Traditional Chinese Medicine of Guangdong Province (grant number 20201025).

## CONFLICT OF INTEREST

The authors declare that they have no competing interests.

## AUTHOR CONTRIBUTIONS

**Zixuan Hu:** Conceptualization (equal); Investigation (equal); Visualization (equal). **Yijing Lai:** Data curation (equal); Investigation (equal); Visualization (equal); Writing‐review & editing (equal). **Chaoya Ma:** Investigation (equal); Writing‐review & editing (equal). **Lina Zuo:** Investigation (equal); Validation (equal). **Guanlin Xiao:** Data curation (equal); Investigation (equal). **Haili Gao:** Investigation (equal); Validation (equal). **Biyuan Xie:** Investigation (equal); Validation (equal). **Xuejun Huang:** Investigation (equal); Methodology (equal); Project administration (equal). **Haining Gan:** Data curation (equal); Methodology (equal). **Dane Huang:** Validation (equal); Writing‐review & editing (equal). **Nan Yao:** Writing‐review & editing (equal). **Baoguo Feng:** Resources (equal); Supervision (equal). **JieXia Ru:** Investigation (equal); Methodology (equal). **Yuxing Chen:** Conceptualization (equal); Supervision (equal). **Dake Cai:** Conceptualization (equal); Funding acquisition (equal); Investigation (equal); Writing‐original draft (equal).

## Supporting information

Appendix S1

## Data Availability

The raw data required to reproduce these findings cannot be shared at this time as the data also form part of an ongoing study.

## References

[fsn32636-bib-0001] Abe, J. , & Morrell, C. (2016). Pyroptosis as a regulated form of necrosis: PI+/Annexin V‐/high caspase 1/Low caspase 9 activity in cells = pyroptosis? Circulation Research, 118(10), 1457–1460. 10.1161/CIRCRESAHA.116.308699 27174943 PMC5137941

[fsn32636-bib-0002] Bai, K. C. , & Sheu, F. (2018). A novel protein from edible fungi *Cordyceps militaris* that induces apoptosis. Journal of Food and Drug Analysis, 26(1), 21–30. 10.1016/j.jfda.2016.10.013 29389557 PMC9332670

[fsn32636-bib-0003] Bao, N. , Ou, J. , Li, N. , Zou, P. , Sun, J. , & Chen, L. (2018). Novel anticancer hybrids from diazen‐1‐ium‐1,2‐diolate nitric oxide donor and ROS inducer plumbagin: Design, synthesis and biological evaluations. European Journal of Medicinal Chemistry, 154, 1–8. 10.1016/j.ejmech.2018.04.047 29772386

[fsn32636-bib-0004] Bassett, J. K. , Hodge, A. M. , English, D. R. , Baglietto, L. , Hopper, J. L. , Giles, G. G. , & Severi, G. (2012). Dietary intake of B vitamins and methionine and risk of lung cancer. European Journal of Clinical Nutrition, 66(2), 182–187. 10.1038/ejcn.2011.157 21878960

[fsn32636-bib-0005] Brentnall, M. , Rodriguez‐Menocal, L. , De Guevara, R. L. , Cepero, E. , & Boise, L. H. (2013). Caspase‐9, caspase‐3 and caspase‐7 have distinct roles during intrinsic apoptosis. BMC Cell Biology, 14, 32. 10.1186/1471-2121-14-32 23834359 PMC3710246

[fsn32636-bib-0006] Bub, A. , Malpuech‐Brugère, C. , Orfila, C. , Amat, J. , Arianna, A. , Blot, A. , Di Nunzio, M. , Holmes, M. , Kertész, Z. , Marshall, L. , Nemeth, I. , Ricciardiello, L. , Seifert, S. , Sutulic, S. , Ulaszewska, M. , & Bordoni, A. (2019). A dietary intervention of bioactive enriched foods aimed at adults at risk of metabolic syndrome: Protocol and results from PATHWAY‐27 pilot study. Nutrients, 11(8), 1814. 10.3390/nu11081814 PMC6723599 31390801

[fsn32636-bib-0007] Cai, H. , Li, J. , Gu, B. , Xiao, Y. , Chen, R. , Liu, X. , Xie, X. , & Cao, L. I. (2018). Extracts of *Cordyceps sinensis* inhibit breast cancer cell metastasis via down‐regulation of metastasis‐related cytokines expression. Journal of Ethnopharmacology, 214, 106–112. 10.1016/j.jep.2017.12.012 29253616

[fsn32636-bib-0008] Chen, L.‐L. , Lin, H.‐P. , Zhou, W.‐J. , He, C.‐X. , Zhang, Z.‐Y. , Cheng, Z.‐L. , Song, J.‐B. , Liu, P. , Chen, X.‐Y. , Xia, Y.‐K. , Chen, X.‐F. , Sun, R.‐Q. , Zhang, J.‐Y. , Sun, Y.‐P. , Song, L. , Liu, B.‐J. , Du, R.‐K. , Ding, C. , Lan, F. , Guan, K.‐L. (2018). SNIP1 recruits TET2 to regulate c‐MYC target genes and cellular DNA damage response. Cell Reports, 25(6), 1485–1500.e1484. 10.1016/j.celrep.2018.10.028 30404004 PMC6317994

[fsn32636-bib-0009] Chen, Y. , Smith, M. R. , Thirumalai, K. , & Zychlinsky, A. (1996). A bacterial invasin induces macrophage apoptosis by binding directly to ICE. EMBO Journal, 15(15), 3853–3860. 10.1002/j.1460-2075.1996.tb00759.x PMC452076 8670890

[fsn32636-bib-0010] Cho, S. H. , & Kang, I. C. (2018). The inhibitory effect of Cordycepin on the proliferation of cisplatin‐resistant A549 lung cancer cells. Biochemical and Biophysical Research Communications, 498(3), 431–436. 10.1016/j.bbrc.2018.02.188 29496448

[fsn32636-bib-0011] Chou, S.‐M. , Lai, W.‐J. , Hong, T.‐W. , Lai, J.‐Y. , Tsai, S.‐H. , Chen, Y.‐H. , Yu, S.‐H. , Kao, C.‐H. , Chu, R. , Ding, S.‐T. , Li, T.‐K. , & Shen, T.‐L. (2014). Synergistic property of cordycepin in cultivated *Cordyceps militaris*‐mediated apoptosis in human leukemia cells. Phytomedicine, 21(12), 1516–1524. 10.1016/j.phymed.2014.07.014 25442260

[fsn32636-bib-0012] Choudhary, G. S. , Al‐Harbi, S. , & Almasan, A. (2015). Caspase‐3 activation is a critical determinant of genotoxic stress‐induced apoptosis. Methods in Molecular Biology, 1219, 1–9. 10.1007/978-1-4939-1661-0_1 25308257

[fsn32636-bib-0013] Fathi, N. , Rashidi, G. , Khodadadi, A. , Shahi, S. , & Sharifi, S. (2018). STAT3 and apoptosis challenges in cancer. International Journal of Biological Macromolecules, 117, 993–1001. 10.1016/j.ijbiomac.2018.05.121 29782972

[fsn32636-bib-0014] Finley, L. W. S. (2019). Metabolic signal curbs cancer‐cell migration. Nature, 571(7763), 39–40. 10.1038/d41586-019-01934-9 31263261

[fsn32636-bib-0015] Han, J. Y. , Im, J. , Choi, J. N. , Lee, C. H. , Park, H. J. , Park, D. K. , Yun, C.‐H. , & Han, S. H. (2010). Induction of IL‐8 expression by *Cordyceps militaris* grown on germinated soybeans through lipid rafts formation and signaling pathways via ERK and JNK in A549 cells. Journal of Ethnopharmacology, 127(1), 55–61. 10.1016/j.jep.2009.09.051 19799982

[fsn32636-bib-0016] Hirsch, F. R. , Scagliotti, G. V. , Mulshine, J. L. , Kwon, R. , Curran, W. J. Jr , Wu, Y. L. , & Paz‐Ares, L. (2017). Lung cancer: Current therapies and new targeted treatments. Lancet, 389(10066), 299–311. 10.1016/S0140-6736(16)30958-8 27574741

[fsn32636-bib-0017] Huang, F. , Ni, M. , Chalishazar, M. D. , Huffman, K. E. , Kim, J. , Cai, L. , Shi, X. , Cai, F. , Zacharias, L. G. , Ireland, A. S. , Li, K. , Gu, W. , Kaushik, A. K. , Liu, X. , Gazdar, A. F. , Oliver, T. G. , Minna, J. D. , Hu, Z. , & DeBerardinis, R. J. (2018). Inosine monophosphate dehydrogenase dependence in a subset of small cell lung cancers. Cell Metabolism, 28(3), 369–382.e365. 10.1016/j.cmet.2018.06.005 30043754 PMC6125205

[fsn32636-bib-0018] Huang, H. , Li, T. , Chen, M. , Liu, F. , Wu, H. , Wang, J. , Chen, J. , & Li, X. I. (2018). Identification and validation of NOLC1 as a potential target for enhancing sensitivity in multidrug resistant non‐small cell lung cancer cells. Cellular & Molecular Biology Letters, 23, 54. 10.1186/s11658-018-0119-8 30505321 PMC6258490

[fsn32636-bib-0019] Hwang, J. H. , Park, S. J. , Ko, W. G. , Kang, S. M. , Lee, D. B. , Bang, J. , Park, B.‐J. , Wee, C.‐B. , Kim, D. J. , Jang, I.‐S. , & Ko, J. H. (2017). Cordycepin induces human lung cancer cell apoptosis by inhibiting nitric oxide mediated ERK/Slug signaling pathway. American Journal of Cancer Research, 7(3), 417–432. 28401001 PMC5385633

[fsn32636-bib-0020] Ichihara, H. , Kuwabara, K. , & Matsumoto, Y. (2017). Trehalose liposomes suppress the growth of tumors on human lung carcinoma‐bearing mice by induction of apoptosis in vivo. Anticancer Research, 37(11), 6133–6139. 10.21873/anticanres.12062 29061794

[fsn32636-bib-0021] Jorgensen, I. , Zhang, Y. , Krantz, B. A. , & Miao, E. A. (2016). Pyroptosis triggers pore‐induced intracellular traps (PITs) that capture bacteria and lead to their clearance by efferocytosis. Journal of Experimental Medicine, 213(10), 2113–2128. 10.1084/jem.20151613 PMC5030797 27573815

[fsn32636-bib-0022] Kamiya, H. , Kanno, T. , Fujita, Y. , Gotoh, A. , Nakano, T. , & Nishizaki, T. (2012). Apoptosis‐related gene transcription in human A549 lung cancer cells via A(3) adenosine receptor. Cellular Physiology and Biochemistry, 29(5–6), 687–696. 10.1159/000312589 22613969

[fsn32636-bib-0023] Kang, R. , Zeng, L. , Zhu, S. , Xie, Y. , Liu, J. , Wen, Q. , Cao, L. , Xie, M. , Ran, Q. , Kroemer, G. , Wang, H. , Billiar, T. R. , Jiang, J. , & Tang, D. (2018). Lipid peroxidation drives gasdermin D‐mediated pyroptosis in lethal polymicrobial sepsis. Cell Host & Microbe, 24(1), 97–108.e104. 10.1016/j.chom.2018.05.009 29937272 PMC6043361

[fsn32636-bib-0024] Kim, Y. S. , Kim, E. K. , Hwang, J. W. , Kim, J. S. , Kim, H. , Dong, X. , Natarajan, S. B. , Moon, S.‐H. , Jeon, B.‐T. , & Park, P. J. (2017). Fermented Asterina pectinifera with *Cordyceps militaris* mycelia induced apoptosis in B16F10 melanoma cells. Advances in Experimental Medicine and Biology, 975(Pt 2), 1141–1152. 10.1007/978-94-024-1079-2_91 28849529

[fsn32636-bib-0025] Ko, C. H. , Shen, S. C. , & Chen, Y. C. (2004). Hydroxylation at C4' or C6 is essential for apoptosis‐inducing activity of flavanone through activation of the caspase‐3 cascade and production of reactive oxygen species. Free Radical Biology and Medicine, 36(7), 897–910. 10.1016/j.freeradbiomed.2003.12.020 15019974

[fsn32636-bib-0026] Kovacs, S. B. , & Miao, E. A. (2017). Gasdermins: Effectors of pyroptosis. Trends in Cell Biology, 27(9), 673–684. 10.1016/j.tcb.2017.05.005 28619472 PMC5565696

[fsn32636-bib-0027] Lamkanfi, M. , & Kanneganti, T. D. (2010). Caspase‐7: A protease involved in apoptosis and inflammation. International Journal of Biochemistry & Cell Biology, 42(1), 21–24. 10.1016/j.biocel.2009.09.013 19782763 PMC2787741

[fsn32636-bib-0028] Lee, H. , Kim, Y. J. , Kim, H. W. , Lee, D. H. , Sung, M. K. , & Park, T. (2006). Induction of apoptosis by *Cordyceps militaris* through activation of caspase‐3 in leukemia HL‐60 cells. Biological and Pharmaceutical Bulletin, 29(4), 670–674. 10.1248/bpb.29.670 16595897

[fsn32636-bib-0029] Lee, H. H. , Lee, S. , Lee, K. , Shin, Y. S. , Kang, H. , & Cho, H. (2015). Anti‐cancer effect of *Cordyceps militaris* in human colorectal carcinoma RKO cells via cell cycle arrest and mitochondrial apoptosis. DARU Journal of Pharmaceutical Sciences, 23, 35. 10.1186/s40199-015-0117-6 26141646 PMC4491205

[fsn32636-bib-0030] Lee, J. S. , & Hong, E. K. (2011). Immunostimulating activity of the polysaccharides isolated from *Cordyceps militaris* . International Immunopharmacology, 11(9), 1226–1233. 10.1016/j.intimp.2011.04.001 21497206

[fsn32636-bib-0031] Lehmler, H. J. , Xu, L. , Vyas, S. M. , Ojogun, V. A. , Knutson, B. L. , & Ludewig, G. (2008). Synthesis, physicochemical properties and in vitro cytotoxicity of nicotinic acid ester prodrugs intended for pulmonary delivery using perfluorooctyl bromide as vehicle. International Journal of Pharmaceutics, 353(1–2), 35–44. 10.1016/j.ijpharm.2007.11.011 18164563 PMC2408535

[fsn32636-bib-0032] Lei, Q. , Yi, T. , & Chen, C. (2018). NF‐kappaB‐Gasdermin D (GSDMD) axis couples oxidative stress and NACHT, LRR and PYD domains‐containing protein 3 (NLRP3) inflammasome‐mediated cardiomyocyte pyroptosis following myocardial infarction. Medical Science Monitor, 24, 6044–6052. 10.12659/MSM.908529 30161099 PMC6128186

[fsn32636-bib-0033] Li, X. Y. , Tao, H. , Jin, C. , Du, Z. Y. , Liao, W. F. , Tang, Q. J. , & Ding, K. (2020). Cordycepin inhibits pancreatic cancer cell growth in vitro and in vivo via targeting FGFR2 and blocking ERK signaling. Chinese Journal of Natural Medicines, 18(5), 345–355. 10.1016/S1875-5364(20)30041-8 32451092

[fsn32636-bib-0034] Liang, J. , Zhou, J. , Xu, Y. , Huang, X. , Wang, X. , Huang, W. , & Li, H. (2020). Osthole inhibits ovarian carcinoma cells through LC3‐mediated autophagy and GSDME‐dependent pyroptosis except for apoptosis. European Journal of Pharmacology, 874, 172990. 10.1016/j.ejphar.2020.172990 32057718

[fsn32636-bib-0035] Makimoto, G. , Ichihara, E. , Hotta, K. , Ninomiya, K. , Oze, I. , Minami, D. , Ninomiya, T. , Kubo, T. , Ohashi, K. , Tabata, M. , Maeda, Y. , & Kiura, K. (2018). Randomized phase II study comparing mannitol with furosemide for the prevention of renal toxicity induced by cisplatin‐based chemotherapy with short‐term low‐volume hydration in advanced non‐small cell lung cancer: The OLCSG1406 study protocol. Acta Medica Okayama, 72(3), 319–323. 10.18926/AMO/56080 29926012

[fsn32636-bib-0036] McKibbin, T. , Cheng, L. L. , Kim, S. , Steuer, C. E. , Owonikoko, T. K. , Khuri, F. R. , Shin, D. M. , & Saba, N. F. (2016). Mannitol to prevent cisplatin‐induced nephrotoxicity in patients with squamous cell cancer of the head and neck (SCCHN) receiving concurrent therapy. Supportive Care in Cancer, 24(4), 1789–1793. 10.1007/s00520-015-2978-0 26446698

[fsn32636-bib-0037] Merk, J. , Rolff, J. , Dorn, C. , Leschber, G. , & Fichtner, I. (2011). Chemoresistance in non‐small‐cell lung cancer: Can multidrug resistance markers predict the response of xenograft lung cancer models to chemotherapy? European Journal of Cardio‐Thoracic Surgery, 40(1), e29–e33. 10.1016/j.ejcts.2011.02.010 21420313

[fsn32636-bib-0038] Nargi‐Aizenman, J. L. , Simbulan‐Rosenthal, C. M. , Kelly, T. A. , Smulson, M. E. , & Griffin, D. E. (2002). Rapid activation of poly(ADP‐ribose) polymerase contributes to Sindbis virus and staurosporine‐induced apoptotic cell death. Virology, 293(1), 164–171. 10.1006/viro.2001.1253 11853409

[fsn32636-bib-0039] Nasser, M. I. , Masood, M. , Wei, W. , Li, X. , Zhou, Y. , Liu, B. , Li, J. , & Li, X. (2017). Cordycepin induces apoptosis in SGC7901 cells through mitochondrial extrinsic phosphorylation of PI3K/Akt by generating ROS. International Journal of Oncology, 50(3), 911–919. 10.3892/ijo.2017.3862 28197639

[fsn32636-bib-0040] Peng, Z. , Wang, P. , Song, W. , Yao, Q. , Li, Y. , Liu, L. , Li, Y. , & Zhou, S. (2020). GSDME enhances Cisplatin sensitivity to regress non‐small cell lung carcinoma by mediating pyroptosis to trigger antitumor immunocyte infiltration. Signal Transduction and Targeted Therapy, 5(1), 159. 10.1038/s41392-020-00274-9 32839451 PMC7445264

[fsn32636-bib-0041] Pietrzak, J. , Spickett, C. M. , Ploszaj, T. , Virag, L. , & Robaszkiewicz, A. (2018). PARP1 promoter links cell cycle progression with adaptation to oxidative environment. Redox Biology, 18, 1–5. 10.1016/j.redox.2018.05.017 29886395 PMC5991907

[fsn32636-bib-0042] Qiao, J. , Shuai, Y. , Zeng, X. , Xu, D. , Rao, S. , Zeng, H. , & Li, F. (2019). Comparison of chemical compositions, bioactive ingredients, and in vitro antitumor activity of four products of cordyceps (ascomycetes) strains from China. International Journal of Medicinal Mushrooms, 21(4), 331–342. 10.1615/IntJMedMushrooms.2019030329 31002629

[fsn32636-bib-0043] Quan, X. , Kwak, B. S. , Lee, J. Y. , Park, J. H. , Lee, A. , Kim, T. H. , & Park, S. (2020). *Cordyceps militaris* induces immunogenic cell death and enhances antitumor immunogenic response in breast cancer. Evidence‐Based Complementary and Alternative Medicine, 2020, 9053274. 10.1155/2020/9053274 32963576 PMC7486645

[fsn32636-bib-0044] Ramirez, M. L. G. , Poreba, M. , Snipas, S. J. , Groborz, K. , Drag, M. , & Salvesen, G. S. (2018). Extensive peptide and natural protein substrate screens reveal that mouse caspase‐11 has much narrower substrate specificity than caspase‐1. Journal of Biological Chemistry, 293(18), 7058–7067. 10.1074/jbc.RA117.001329 PMC5936834 29414788

[fsn32636-bib-0045] Samanta, S. , Zhou, Z. , Rajasingh, S. , Panda, A. , Sampath, V. , & Rajasingh, J. (2018). DNMT and HDAC inhibitors together abrogate endotoxemia mediated macrophage death by STAT3‐JMJD3 signaling. International Journal of Biochemistry & Cell Biology, 102, 117–127. 10.1016/j.biocel.2018.07.002 30010012 PMC6309960

[fsn32636-bib-0046] Sansone, C. , Bruno, A. , Piscitelli, C. , Baci, D. , Fontana, A. , Brunet, C. , Noonan, D. M. , & Albini, A. (2021). Natural compounds of marine origin as inducers of immunogenic cell death (ICD): Potential role for cancer interception and therapy. Cells, 10(2), 231. 10.3390/cells10020231 33504012 PMC7912082

[fsn32636-bib-0047] Sartini, D. , Seta, R. , Pozzi, V. , Morganti, S. , Rubini, C. , Zizzi, A. , Tomasetti, M. , Santarelli, L. , & Emanuelli, M. (2015). Role of nicotinamide N‐methyltransferase in non‐small cell lung cancer: In vitro effect of shRNA‐mediated gene silencing on tumourigenicity. Biological Chemistry, 396(3), 225–234. 10.1515/hsz-2014-0231 25204218

[fsn32636-bib-0048] Sborgi, L. , Ruhl, S. , Mulvihill, E. , Pipercevic, J. , Heilig, R. , Stahlberg, H. , Farady, C. , Müller, D. , Broz, P. , & Hiller, S. (2016). GSDMD membrane pore formation constitutes the mechanism of pyroptotic cell death. EMBO Journal, 35(16), 1766–1778. 10.15252/embj.201694696 PMC5010048 27418190

[fsn32636-bib-0049] Shalini, S. , Dorstyn, L. , Dawar, S. , & Kumar, S. (2015). Old, new and emerging functions of caspases. Cell Death and Differentiation, 22(4), 526–539. 10.1038/cdd.2014.216 25526085 PMC4356345

[fsn32636-bib-0050] Shi, J. , Gao, W. , & Shao, F. (2017). Pyroptosis: Gasdermin‐mediated programmed necrotic cell death. Trends in Biochemical Sciences, 42(4), 245–254. 10.1016/j.tibs.2016.10.004 27932073

[fsn32636-bib-0051] Swartz, M. D. , Peterson, C. B. , Lupo, P. J. , Wu, X. , Forman, M. R. , Spitz, M. R. , Hernandez, L. M. , Vannucci, M. , & Shete, S. (2013). Investigating multiple candidate genes and nutrients in the folate metabolism pathway to detect genetic and nutritional risk factors for lung cancer. PLoS One, 8(1), e53475. 10.1371/journal.pone.0053475 23372658 PMC3553105

[fsn32636-bib-0052] Wang, W. , Zhu, M. , Xu, Z. , Li, W. , Dong, X. U. , Chen, Y. I. , Lin, B. O. , & Li, M. (2019). Ropivacaine promotes apoptosis of hepatocellular carcinoma cells through damaging mitochondria and activating caspase‐3 activity. Biological Research, 52(1), 36. 10.1186/s40659-019-0242-7 31300048 PMC6625015

[fsn32636-bib-0053] Wang, Y. , Gao, W. , Shi, X. , Ding, J. , Liu, W. , He, H. , Wang, K. , & Shao, F. (2017). Chemotherapy drugs induce pyroptosis through caspase‐3 cleavage of a gasdermin. Nature, 547(7661), 99–103. 10.1038/nature22393 28459430

[fsn32636-bib-0054] Xu, L. , Fan, Q. , Wang, X. , Zhao, X. , & Wang, L. (2016). Inhibition of autophagy increased AGE/ROS‐mediated apoptosis in mesangial cells. Cell Death & Disease, 7(11), e2445. 10.1038/cddis.2016.322 27809300 PMC5260901

[fsn32636-bib-0055] Yang, D. , Song, J. , Wu, L. , Ma, Y. , Song, C. , Dovat, S. , Nishizaki, T. , & Liu, J. (2013). Induction of senescence by adenosine suppressing the growth of lung cancer cells. Biochemical and Biophysical Research Communications, 440(1), 62–67. 10.1016/j.bbrc.2013.09.030 24051088

[fsn32636-bib-0056] Yang, X. , Cheng, X. , Tang, Y. , Qiu, X. , Wang, Y. , Kang, H. , Wu, J. , Wang, Z. , Liu, Y. , Chen, F. , Xiao, X. , Mackman, N. , Billiar, T. R. , Han, J. , & Lu, B. (2019). Bacterial endotoxin activates the coagulation cascade through gasdermin D‐dependent phosphatidylserine exposure. Immunity, 51(6), 983–996.e986. 10.1016/j.immuni.2019.11.005 31836429

[fsn32636-bib-0057] Yang, Y. , Karakhanova, S. , Hartwig, W. , D'Haese, J. G. , Philippov, P. P. , Werner, J. , & Bazhin, A. V. (2016). Mitochondria and mitochondrial ROS in cancer: Novel targets for anticancer therapy. Journal of Cellular Physiology, 231(12), 2570–2581. 10.1002/jcp.25349 26895995

[fsn32636-bib-0058] Yu, J. , Li, S. , Qi, J. , Chen, Z. , Wu, Y. , Guo, J. , Wang, K. , Sun, X. , & Zheng, J. (2019). Cleavage of GSDME by caspase‐3 determines lobaplatin‐induced pyroptosis in colon cancer cells. Cell Death & Disease, 10(3), 193. 10.1038/s41419-019-1441-4 30804337 PMC6389936

[fsn32636-bib-0059] Zhang, C.‐C. , Li, C.‐G. , Wang, Y.‐F. , Xu, L.‐H. , He, X.‐H. , Zeng, Q.‐Z. , Zeng, C.‐Y. , Mai, F.‐Y. , Hu, B. O. , & Ouyang, D.‐Y. (2019). Chemotherapeutic paclitaxel and cisplatin differentially induce pyroptosis in A549 lung cancer cells via caspase‐3/GSDME activation. Apoptosis, 24(3–4), 312–325. 10.1007/s10495-019-01515-1 30710195

[fsn32636-bib-0060] Zhang, C. , Zhong, Q. , Zhang, X. F. , Hu, D. X. , He, X. M. , Li, Q. L. , & Feng, T. (2015). Effects of cordycepin on proliferation, apoptosis and NF‐kappaB signaling pathway in A549 cells. Zhong Yao Cai, 38(4), 786–789. 26672348

[fsn32636-bib-0061] Zhang, L. , Liu, X. , Tang, Z. , Li, X. , & Wang, G. (2016). Reversal of galectin‐1 gene silencing on resistance to cisplatin in human lung adenocarcinoma A549 cells. Biomedicine & Pharmacotherapy, 83, 265–270. 10.1016/j.biopha.2016.06.030 27392028

[fsn32636-bib-0062] Zhaolin, Z. , Jiaojiao, C. , Peng, W. U. , Yami, L. , Tingting, Z. , Jun, T. , Shiyuan, W. U. , Jinyan, X. , Dangheng, W. , Zhisheng, J. , & Zuo, W. (2019). OxLDL induces vascular endothelial cell pyroptosis through miR‐125a‐5p/TET2 pathway. Journal of Cellular Physiology, 234(5), 7475–7491. 10.1002/jcp.27509 30370524

[fsn32636-bib-0063] Zhu, M. , Wang, J. , Xie, J. , Chen, L. , Wei, X. , Jiang, X. , Bao, M. , Qiu, Y. , Chen, Q. , Li, W. , Jiang, C. , Zhou, X. , Jiang, L. , Qiu, P. , & Wu, J. (2018). Design, synthesis, and evaluation of chalcone analogues incorporate alpha, beta‐Unsaturated ketone functionality as anti‐lung cancer agents via evoking ROS to induce pyroptosis. European Journal of Medicinal Chemistry, 157, 1395–1405. 10.1016/j.ejmech.2018.08.072 30196062

