# Supplementary material for: Cordyceps militaris extract induces apoptosis and pyroptosis via caspase‐3/PARP/GSDME pathways in A549 cell line
Source: Food Sci Nutr. 2021 Oct 30;10(1):21–38. doi: 10.1002/fsn3.2636 (PMC8751435; doi:10.1002/fsn3.2636)
Supplement: Supplementary file 1 — Appendix S1 [file FSN3-10-21-s002.doc]

**Supplement: HPLC assay for** ***Cordyceps Militaris*** **Extract** **(CME)**

1. **Detections for cordycepin and adenosine**

Instrument: Agilent 1260 high performance liquid chromatograph (HPLC) with diode array detector (DAD, 200-400nm).

Column: SGE protecol C18 (250mm×4.6mm, 5μm)

Column temperature: 35℃

Elution program: acetonitrile ：water (5∶95,V/V).

Flow velocity: 1mL/min

Detection wave: 260nm (Figure 1S);

Sample volume: 20μl

Figure 1S HPLC identification of cordycepin and adenosine in *Cordyceps Militaris* Extract ( CME ).

1. **Determination for** **cordycepin and** **adenosine in CME by HPLC.**

Contents of cordycepin and adenosine are 3749.29±36.90 mg/kg,4022.26±13.58 mg/kg respectively in CME.(Figure 2S)

**
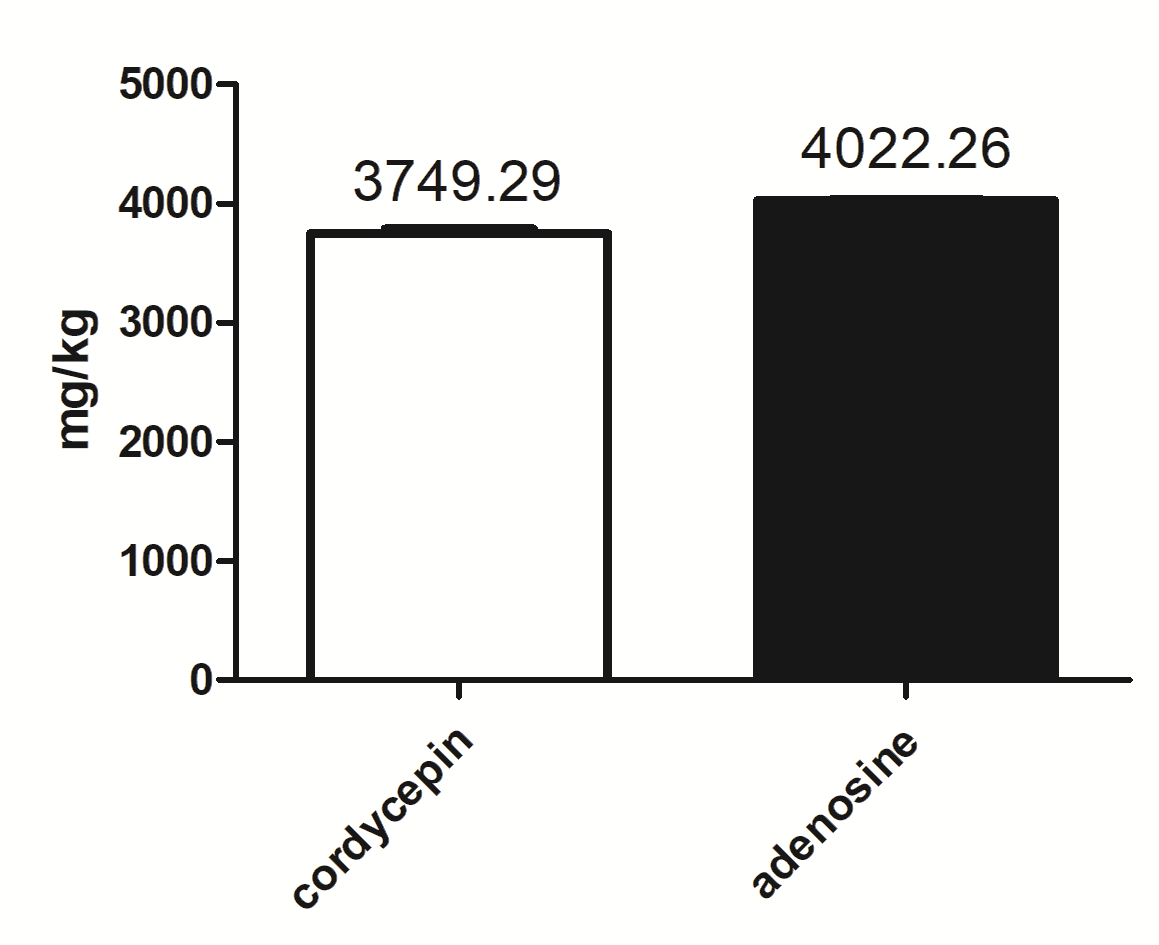
**

Figure 2S Contents of cordycepin and adenosine in CME.
