# Supplementary figures and images for: Cordyceps militaris extract induces apoptosis and pyroptosis via caspase‐3/PARP/GSDME pathways in A549 cell line
Source: Food Sci Nutr. 2021 Oct 30;10(1):21–38. doi: 10.1002/fsn3.2636 (PMC8751435; doi:10.1002/fsn3.2636)

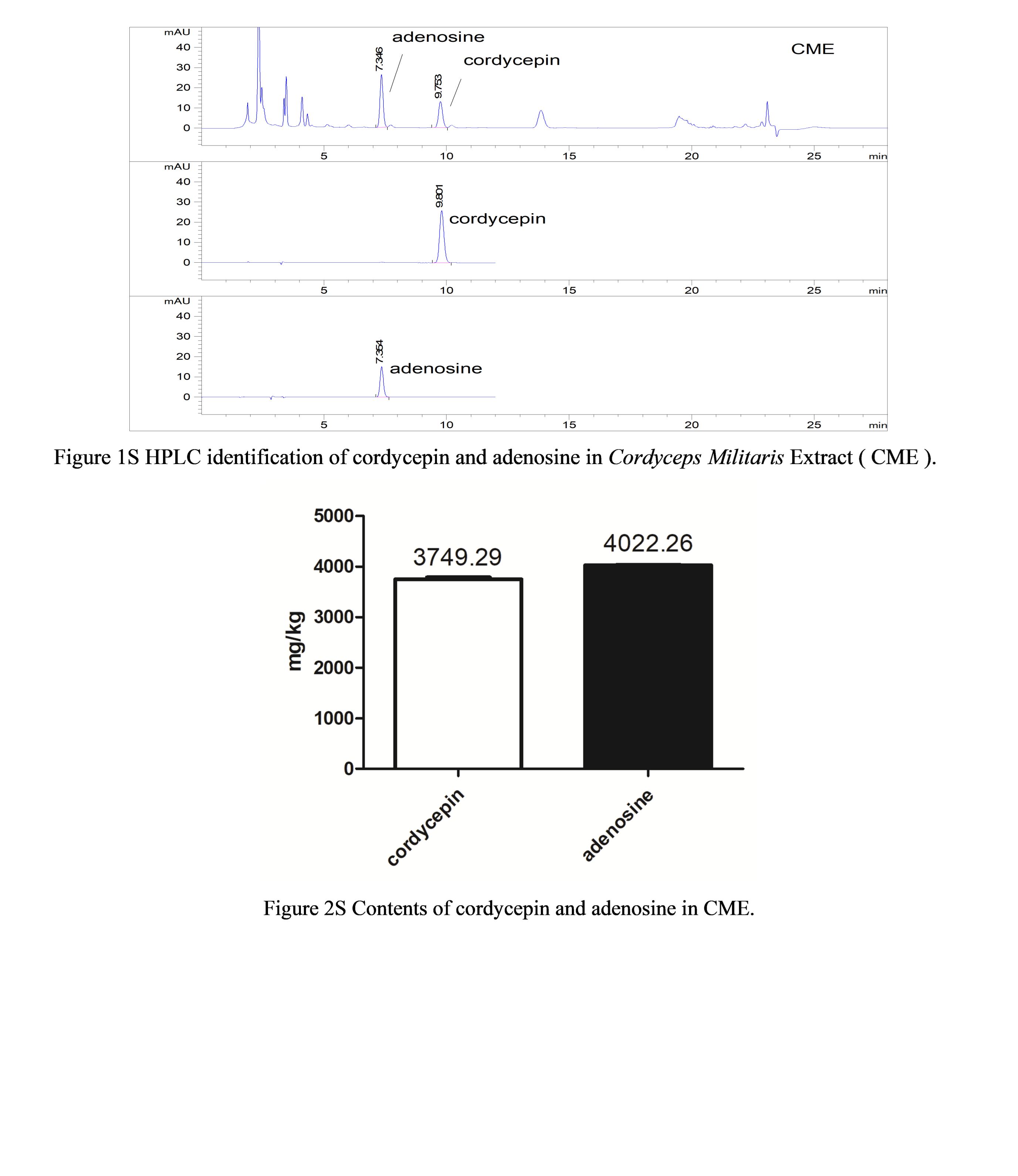

Supplement: Supplementary file 2 [file FSN3-10-21-s001.tif]
